# Synthesis of diaryl phosphates using phytic acid as a phosphorus source

**DOI:** 10.3762/bjoc.22.15

**Published:** 2026-01-30

**Authors:** Kazuya Asao, Seika Matsumoto, Haruka Mori, Riku Yoshimura, Takeshi Sasaki, Naoya Hirata, Yasuyuki Hayakawa, Shin-ichi Kawaguchi

**Affiliations:** 1 The United Graduate School of Agricultural Sciences, Kagoshima University, 1-21-24 Korimoto, Kagoshima City, Kagoshima 890-0065, Japan; 2 Center for Bioresource Education and Research, Saga University, 152-1 Shonan-cho, Karatsu, Saga 847-0021, Japan; 3 Nippon Concrete Industries Co., Ltd., 4-6-14 Shibaura, Minato-ku, Tokyo 108-8560, Japan

**Keywords:** diaryl phosphates, phosphate esters, phosphate ester synthesis, phosphorus recovery, phytic acid

## Abstract

Phytic acid is a phosphorus-rich molecule, which is produced by plants using water-soluble phosphates absorbed from soil. It can potentially serve as a phosphorus source in the syntheses of organic phosphates; however, this approach has not been utilized for the preparation of phosphate esters. In this study, we report the first successful synthesis of phosphate esters using phytic acid as a phosphorus source. Crude products of phosphate diesters were obtained through the reactions of commercially available phytic acid and aromatic alcohols with ^31^P nuclear magnetic resonance yields up to 83%. We also isolated a portion of the reaction substrates with yields up to 60%. Next, we extracted phytic acid from rice bran with a recovery of 4.2% and then conducted an esterification reaction using the extracted phytic acid and phenol. As a result, diphenyl phosphate with a yield of 44% was obtained. This work can facilitate the development of an environmentally friendly method for producing phosphate esters that does not rely on phosphate rock but instead uses biomass as a phosphorus source.

## Introduction

**Scheme 1:**
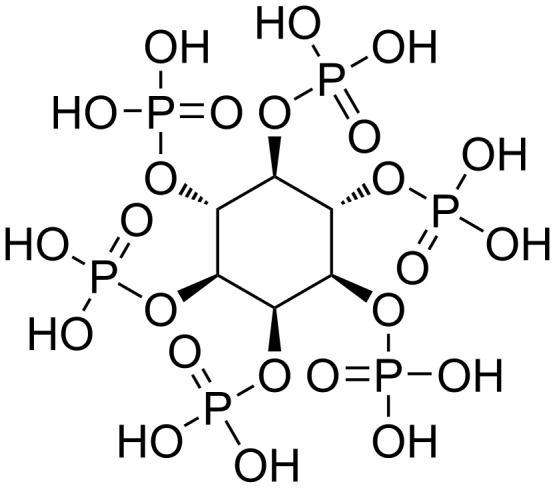
Structure of phytic acid.

Phytic acid (*myo*-inositol-1,2,3,4,5,6-hexakisphosphate, Scheme 1) is a phosphorus-rich molecule, which is produced by plants using water-soluble phosphates absorbed from soil and stored as a phosphorus source in their bodies. Rice bran accounts for 10% of rice weight and contains approximately 6 g phytic acid/100 g rice bran [1–3]. Phytic acid has a structure, in which six phosphoric acid molecules condense into a *myo*-inositol skeleton. Phytic acid can be enzymatically hydrolyzed in the presence of phytases, resulting in the release of phosphoric acid molecules [3–5]. Plants and microorganisms possess their own phytases and thus utilize phytic acid as a phosphorus source. However, the artificial hydrolysis of phytic acid without phytases is extremely difficult because of its hydrolysis resistance, and only a few studies have considered the utilization of phytic acid as a phosphorus source [6]. In recent years, industrial applications of phytic acid have been investigated for its potential utilization in metal-chelating agents, flame retardants, and catalysts [3,6–14]. However, these applications involved the use of either phytic acid itself or its derivatives.

**Figure 1:**
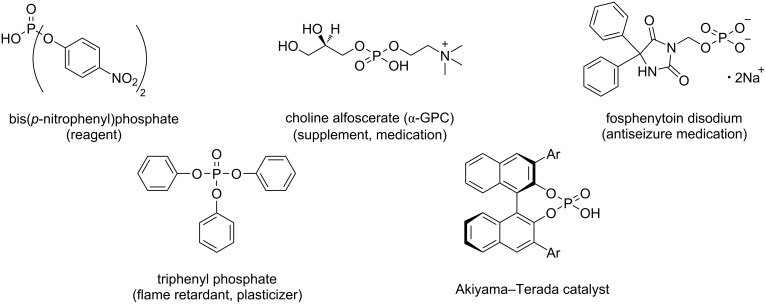
Structures of representative phosphate esters.

Our group has previously reported a method for synthesizing diaryl phosphates using phosphoric acid as the phosphorus source [15]. There are three types of phosphate esters – monoesters, diesters, and triesters – which differ in their physical properties, reactivities, and practical applications (Figure 1). Phosphate monoesters and diesters are commonly found in biomolecules such as nucleobases and adenosine phosphates [16]. Drugs and reagents containing phosphate ester moieties were designed as analogs of these molecules [16–20]. Phosphate monoesters with long aliphatic chains have also been used as surfactants [21]. Phosphate triesters are utilized as flame retardants and plasticizers for polymers [22] and as insecticides [23]. Particularly, phosphate diesters are among the most important synthetic catalysts, such as the Akiyama–Terada catalyst that serves as a chiral Brønsted acid catalyst [24–25] and promotes a variety of stereoselective reactions. Phosphate diesters also catalyze the ring-opening polymerization of some lactones [26–27] and are important for the syntheses of ionic liquids [28–29] and asymmetric phosphate esters. Furthermore, a novel metal extractant based on diaryl phosphate has recently been reported [30–31].

**Figure 2:**
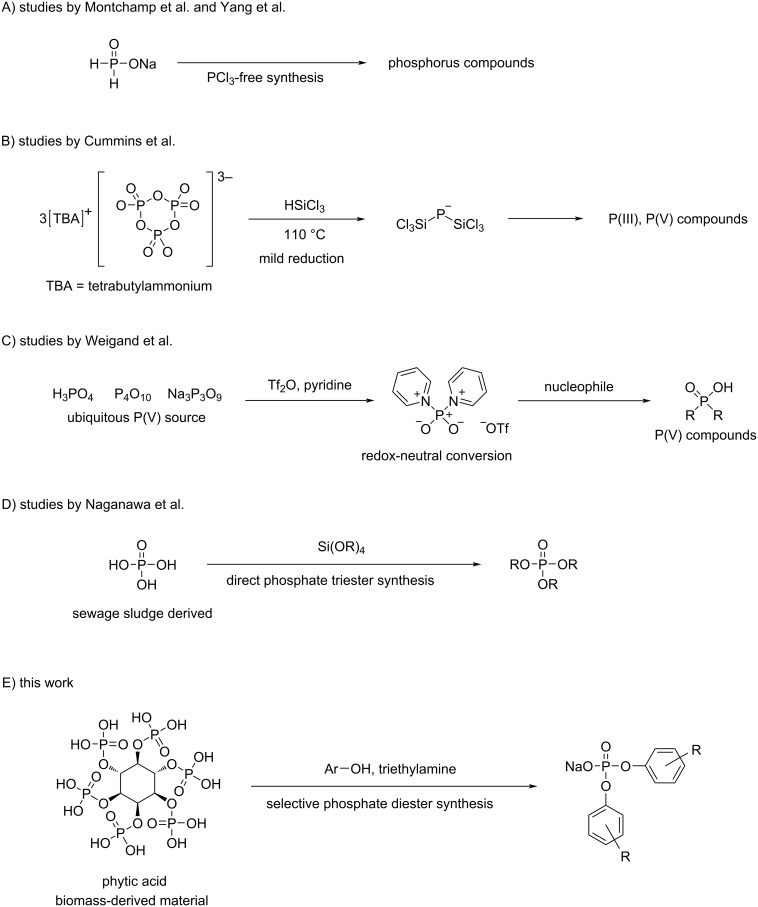
Synthetic methods for phosphorus compounds bypassing white phosphorus or phosphorus chloride.

Phosphate esters are generally synthesized from phosphoryl chlorides and the corresponding alcohols, which generates large amounts of heat and hydrogen chloride gas [32–33]. Focusing on the upstream material, phosphoryl chlorides are obtained via the chlorination of white phosphorus and excess chlorine gas. Further upstream, white phosphorus is obtained from phosphate rocks, a practice that has evoked concerns due to the natural resource depletion through a refining process called the Wöhler process [34]. It incurs extremely high energy costs of approximately 12.5–14 MWh per 1 t (1 metric ton = 1000 kg) of white phosphorus [34]. In recent years, synthetic methods for phosphorus chemicals that do not depend on white phosphorus or phosphorus chloride have been developed to reduce energy consumption and environmental hazards. For example, Montchamp’s group and Yang’s group have focused on using sodium phosphinate as an alternative to PCl_3_, and various halogen-free synthetic methods have been reported for the synthesis of phosphorus compounds (Figure 2A) [35–41]. Cummins’s group demonstrated that phosphoric acid and condensed phosphoric acid can be reduced using trichlorosilane. The resulting intermediate, the bis(trichlorosilyl)phosphide anion [P(SiCl_3_)_2_]^−^, can be converted into a variety of phosphorus compounds, including P(III) species, demonstrating the potential to reduce reliance on white phosphorus (Figure 2B) [42–43]. In addition, Weigand’s group reported a method using Tf_2_O and pyridine to convert P(V) compounds such as phosphoric acid and condensed phosphoric acid into a versatile PO^2+^ phosphorylation agent, (pyridine)_2_PO_2_[OTf]. The intermediate reacts with a variety of nucleophiles, providing a redox-neutral method for the flexible synthesis of P(V) compounds (Figure 2C) [44]. Furthermore, Naganawa’s group recently achieved the direct triesterification of phosphoric acid using organosilicates, and also demonstrated that the reaction can be performed using crude phosphoric acid recovered from sewage sludge ash (Figure 2D) [45].

Our aim was to synthesize phosphate esters, which are essential industrial materials, via esterification with alcohols using sustainable biomass-derived phytic acid as a phosphorus source (Figure 2E). This approach can facilitate the development of environmentally friendly phosphate ester production as well as phosphorus recovery and recycling techniques. To date, the preparation of phosphate esters utilizing phytic acid as a phosphorus source has not been realized yet. Herein, we examined the syntheses of diaryl phosphates using commercially available phytic acid and phytic acid extracted from rice bran.

## Results and Discussion

### Synthesis of diaryl phosphates using commercial phytic acid

**Figure 3:**
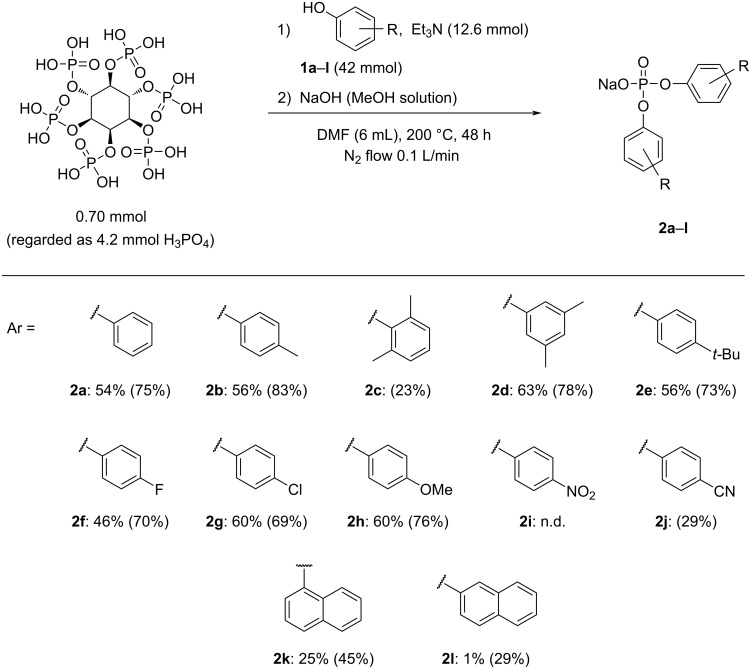
Conditions for the syntheses of diaryl phosphates using phytic acid as a phosphorus source and scope of substrate applications. Each diaryl phosphate was isolated as a sodium salt, and the isolated yields are listed below. The yields in parentheses represent ^31^P nuclear magnetic resonance (NMR) yields defined based on a calibration curve using triphenylphosphine oxide as an internal standard. The isolation of **2c** and **2j** was not accomplished because of their high hygroscopicity and occurrence of large amounts of by-products; therefore, their identification and reaction yield calculation were performed using standards which were prepared via alternative routes (see section 1.4. in Supporting Information File 1). n.d. = not detected.

**Scheme 2:**
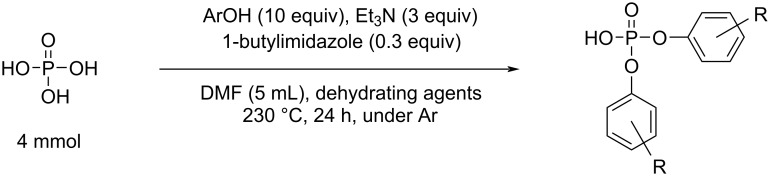
Phosphoric acid diesterification reaction conducted in a previous study.

First, commercially available phytic acid was used instead of phosphoric acid for the syntheses of diaryl phosphates (Figure 3). Phosphate esters were synthesized using the glass apparatus depicted in Figure S1 of Supporting Information File 1. All reactions were performed under a N_2_ flow (0.1 L/min) from a three-way stopcock for water removal. The reaction conditions for this synthesis procedure were based on the results of our previous study dedicated to the esterification of phosphoric acid and phenols [15] (see Scheme 2). Under the optimized reaction conditions in our previous work, the reaction yield of phosphate esters could be slightly improved by using a catalytic amount of 1-butylimidazole; however, in the present work, a catalyst was not employed to simplify the purification step. The reaction temperature in this process should have been set to 230 °C. However, at this temperature, a burnt black material was obtained in the reaction mixture owing to the side reaction of the inositol moiety of phytic acid, which decreased the isolated yields of diaryl phosphates. Therefore, the reaction temperature was set to 200 °C, and the reaction time was extended to 48 h.

Figure 3 shows the reaction conditions and aromatic alcohols **1a–l** participating in the reaction. Phytic acid has six phosphoric acid moieties, therefore we treated it as if it contained six moles of phosphate per mole of phytic acid. All diaryl phosphates were isolated as sodium salts through precipitation in toluene by neutralization with a NaOH methanol solution.

The isolated diaryl phosphates were brownish, likely due to the presence of trace impurities formed during the reaction; however, the NMR data revealed that few impurity peaks remained. All of these compounds are known, and the NMR spectral data were consistent with previously reported data for the corresponding free acids or sodium salts [15,46–48]. According to Figure 3, each diaryl phosphate was isolated with a yield of approximately 60%, except for some derivatives. It was presumed that 2-hydroxy-*m*-xylol (**1c**) having methyl groups at the *ortho*-positions, led to a diminished yield of **2c** in comparison to the other substrates due to steric hindrance. In addition, when **1i** and **1j** were used, the corresponding phosphate ester **2i** was not obtained at all, and **2j** was formed only in a low yield. It is well known that phenols containing strong electron-withdrawing groups can be good leaving groups [49–52], and thus phosphate esters easily undergo transesterification or hydrolysis reactions. Considering these factors, we assumed that shifting the reaction equilibrium of compounds derived from phenols containing strong electron-withdrawing groups toward phosphate diester formation would be difficult. Furthermore, when 1-naphthol (**1k**) and 2-naphthol (**1l**) were used as substrates, the ^31^P NMR yields of the produced diesters were 45% (**2k**) and 29% (**2l**), respectively, and their isolation was difficult because of the presence of large amounts of naphthol-derived by-products. When the reactions were performed using alkyl alcohols, their behavior differed significantly from that of aromatic alcohols, and phosphate diesters were not obtained selectively.

**Scheme 3:**
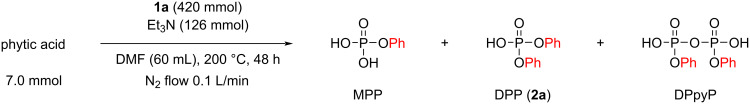
Scale-up reaction conducted for the time-course analysis of phosphate esterification. The reaction apparatus included an oil bath, and the N_2_ flow was set to 0.1 L/min. Phytic acid (7.0 mmol, 50 wt % aqueous solution) was added to dimethylformamide (DMF, 60 mL), after which triethylamine (126 mmol) and **1a** (420 mmol) were poured into the reaction mixture.

**Figure 4:**
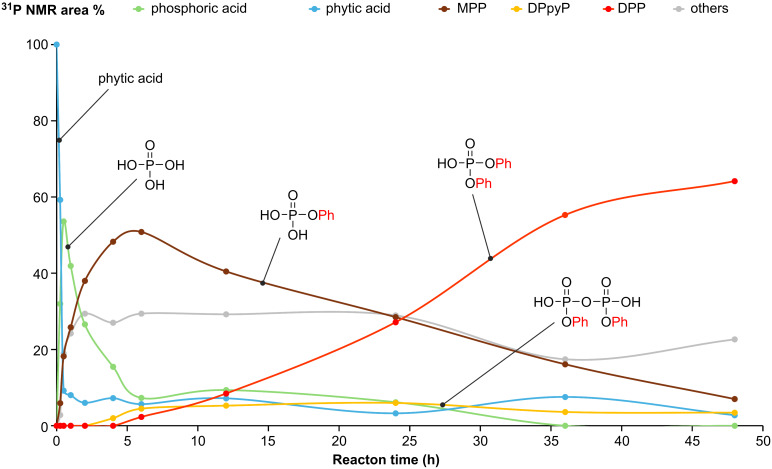
Time-course analysis plots of the diphenylphosphate formation from phytic acid and **1a**. The beginning of the reaction was determined from the time when the oil bath temperature reached 200 °C, and the reaction was performed for up to 48 h under mixing with a magnetic stirrer. The reaction mixture was sampled at certain times and analyzed via ^31^P NMR. Abbreviations: MPP, monophenyl phosphate; DPpyP, diphenyl pyrophosphoric acid; DPP, diphenyl phosphate.

According to the results of the esterification time-course analysis via ^31^P NMR presented in Scheme 3 as well as in Figure 4 and Figure S2 in Supporting Information File 1, phytic acid decomposed immediately after the oil bath temperature increased to 200 °C and released phosphoric acid into the reaction mixture. We assume that two types of phytic acid degradation occurred. One is a simple hydrolysis of the phosphate ester bond, and the other mechanism is based on an E2 reaction, which involves the elimination of phosphoric acid accompanied by alkene formation. The phosphoric acid ester of a secondary alcohol releases phosphoric acid and alkenes through thermal degradation, which has been previously observed using isosorbide phosphate by Daniel and Howell [53]. Furthermore, the formation of aryl phosphate esters from phytic acid may occur via phosphate transesterification between phytic acid and phenols. However, recent transesterification studies have mainly focused on the transesterification of phosphate esters with electron-withdrawing leaving groups [49–50,54], and the transesterification of alkyl phosphates has rarely been observed [55].

**Figure 5:**
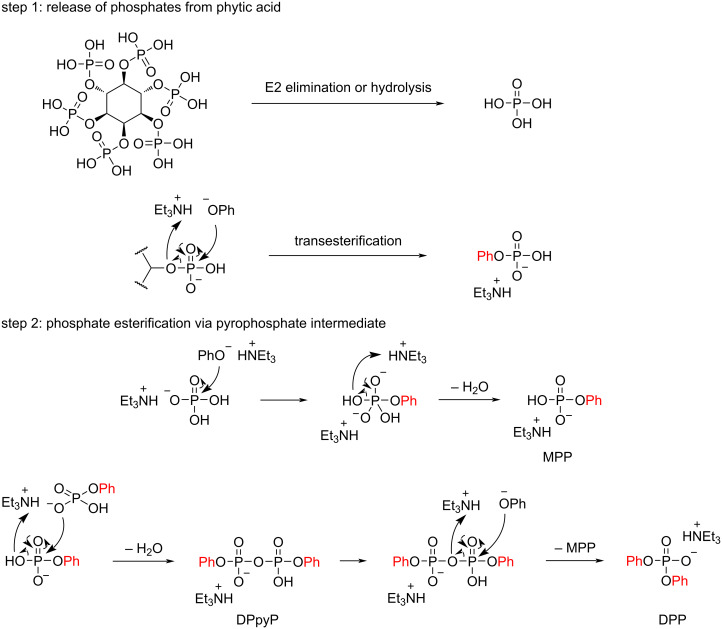
A possible reaction pathway for the formation of phosphate ester using phytic acid as the phosphorus source.

A possible reaction pathway is shown in Figure 5. First, triethylamine increases the solubility of phytic acid in DMF by improving its lipophilicity and accelerates the formation of phosphoric acid through a hydrolysis or E2 elimination reaction. The generated phosphoric acid forms a salt with triethylamine. Next, the phenoxy anion activated by the amine undergoes a nucleophilic attack on the phosphorus atom of the salt of phosphoric acid, resulting in the formation of MPP with the elimination of H_2_O. Then, two MPP molecules undergo a dehydration condensation reaction to form DPpyP [56]. Finally, the phenoxy anion undergoes a nucleophilic attack on the phosphorus atom of DPpyP, and DPP is formed through the elimination of MPP. In the absence of triethylamine, the esterification reaction gives a lower yield because the phytic acid forms a syrup-like precipitate in the reaction mixture due to its poor solubility in organic solvents, thereby preventing efficient release of phosphoric acid into the reaction mixture.

The plot depicted in Figure 4 shows that there is almost no induction period for the formation of the phosphate monoester from phosphoric acid; however, an induction period associated with diester formation is observed between the formation of the monoester and the formation of intermediate DPpyP. This difference suggests that the monoester and the diester are formed via different mechanisms. In addition, the DPpyP ^31^P NMR peak increases and decreases in conjunction with the decrease of the MPP peak and increase of the DPP peak, suggesting that DPpyP acts as an intermediate in the DPP formation process. When alcohol **1c** was used (Figure 3), the reaction stopped mainly at the pyrophosphate ester intermediate because of its steric hindrance, and the yield of diaryl phosphate was low.

### Phytic acid extraction and phosphate ester synthesis

**Figure 6:**
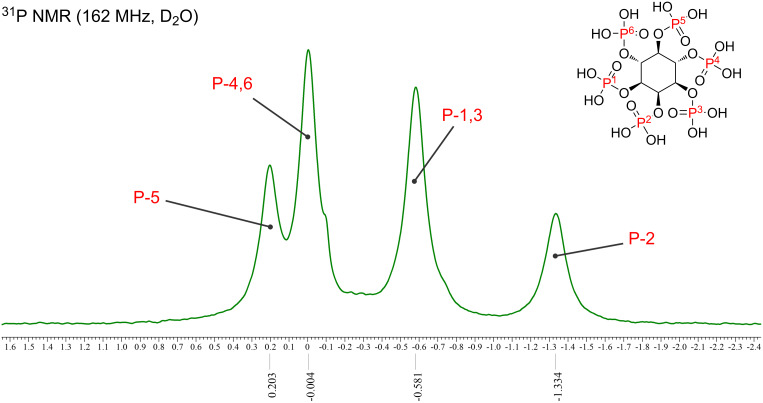
^31^P NMR spectrum of phytic acid extracted from rice bran.

The extraction of phytic acid from rice bran was based on a study conducted by Ida et al. [1]. As rice bran generally contains rice bran oil, it was first delipidated with *n*-hexane. Phytic acid was extracted with 1 M HCl for 1 h. After extraction, the pH value was adjusted to 4.5 with 8.0 M NaOH, which is the isoelectric point of rice bran protein. Solids were removed via filtration and centrifugation followed by supernatant collection. The phytate yield and purity exhibited a trade-off relationship [1], and the yield increased with increasing supernatant pH. Because the phytic acid purity was prioritized in this work, the pH value was increased by the addition of 1.5 M Na_2_CO_3_ up to pH 7 to form a phytate precipitate. Finally, the collected phytate was dried under vacuum with a yield of 2.52 g per 30 g of rice bran, and the total phosphorus content was 141.3 mg P/1 g phytate. Assuming that one molar equivalent of phytic acid was equal to six molar equivalents of phosphorus, the calculated pure phytic acid content was 1.27 g, resulting in a recovery of 4.2% based on the total P analysis data. Considering the losses that occurred during the purification steps, these results were consistent with the fact that 100 g of rice bran contained approximately 6 g of phytic acid [1–3]. Subsequently, the isolated phytate was desalted using a cation-exchange resin to obtain phytic acid. Cation exchange was performed via a shaking method rather than a column method owing to the low solubility of phytate in water. The ^31^P NMR spectrum of the obtained phytic acid clearly shows four peaks corresponding to the non-equivalent phosphorus atoms in the phytic acid molecule (Figure 6 and Figure S3 in Supporting Information File 1) [57–59]. The full ^1^H, ^13^C, and ^31^P NMR spectra are provided in Supporting Information File 1.

**Scheme 4:**
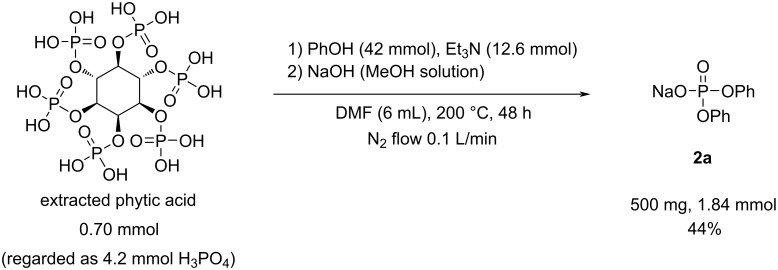
Esterification reaction conditions using the extracted phytic acid. The yield refers to the isolated yield.

Next, the extracted phytic acid was used for phosphate synthesis. Although the reaction was conducted under the same conditions as those utilized for commercial phytic acid (Scheme 4), the reaction mixture became dark, and black precipitates were formed during the reaction. After the distillation of excess phenol and DMF, the insoluble burnt material was removed via filtration. Finally, 500 mg (44% yield) of **2a** was obtained, and its light-brown appearance was identical to that of the product synthesized from commercial phytic acid. High-performance liquid chromatography (HPLC) analysis results indicated that the purity of **2a** derived from the extracted phytic acid was greater than 95% (Supporting Information File 1, Figure S4).

## Conclusion

Herein, we reported a new method for synthesizing phosphate esters using phytic acid as the phosphorus source. Only a few studies on the utilization of phytic acid as a phosphorus source have been conducted in the past [6]. In the present work, phytic acid was extracted from rice bran with a recovery of 4.2%, and phosphate ester **2a** was synthesized using the extracted phytic acid in a good yield (Scheme 4). Furthermore, we demonstrated that the reaction could be applied to phenol derivatives (Figure 3). The time-course analysis of the esterification process via ^31^P NMR (Scheme 3, Figure 4) revealed that phytic acid decomposed immediately after the beginning of the reaction, and that phytic acid released phosphoric acid into the reaction mixture. Furthermore, DPpyP was formed as an intermediate during the reaction. Note that the industrial production of phosphate esters depends on phosphoryl chloride obtained from phosphate rock using white phosphorus as a raw material; this is accompanied by a large electric energy consumption and emission of large amounts of corrosive gases. In our work, the reaction time and temperature can be further improved; however, the obtained results suggest a novel synthetic route for phosphate esters derived from a significantly underutilized phosphorus source. This approach may facilitate the development of an environmentally friendly phosphate production and phosphorus recovery procedure using biomass as a resource without employing phosphorus chloride or upstream raw materials.

## Experimental

### Chemicals and reagents

All reagents and solvents were purchased from Nacalai Tesque (Kyoto, Japan), Tokyo Chemical Industry (Tokyo, Japan), FUJIFILM Wako Pure Chemical Industries (Osaka, Japan), and Sigma-Aldrich (St. Louis, MO, USA). Phytic acid was obtained from Tokyo Chemical Industry. The rice bran used for phytic acid extraction was purchased from a rice-cleaning mill in Saga, Japan.

### Phytic acid extraction

Phytic acid was extracted as described by Ida et al. [1]. *n*-Hexane (300 mL) was poured into rice bran (30 g) and the mixture was stirred with a magnetic stirrer for 1 h. The delipidated rice bran was filtered through a non-woven filter and dried in air overnight. A 1.0 M HCl solution (300 mL) was poured into the rice bran, and the produced mixture was stirred with a magnetic stirrer for 1 h. After stirring, the pH of the extract was adjusted to 4.5 with 8.0 M NaOH, and the extract was filtered using a non-woven filter. The filtrate was centrifuged (2000*g* for 10 min), and the supernatant was collected. The supernatant pH was slowly adjusted to 7.0 with 1.5 M Na_2_CO_3_ to form a sodium phytate precipitate. After the pH reached 7.0, the suspension was left overnight. The next day, the suspension was centrifuged (2000*g*, 10 min), and the supernatant was removed. The remaining pellets were resuspended in an appropriate amount of methanol, and the suspension was filtered via vacuum filtration using ADVANTEC No. 2 filter paper (ADVANTEC TOYO Co. Ltd., Tokyo, Japan). The residue was washed with methanol and dried under vacuum overnight. Finally, 2.52 g of the crude sodium phytate was obtained as a white solid.

### Purification of extracted phytic acid

Well-conditioned DOWEX 50W × 2 100–200 mesh (H) cation exchange resin (Dow Chemical Co., Midland, MI, USA) (60 mL) was poured into deionized water, and crude phytate powder (2.00 g) was added to the suspension. The resulting suspension was stirred for 1 h with a magnetic stirrer. After 1 h, the suspension was poured into a column equipped with a glass filter and eluted with deionized water. The pH of each fraction was measured using pH test paper, and the acidic fractions (pH < 3) were collected. The collected fractions were concentrated to 2–3 mL using a rotary evaporator, and 3.64 g of the extracted phytic acid solution with a total P content of 88.43 mg/mL (measured as described below) was obtained.

### Total P analysis

A total P analysis was performed according to a test method for industrial wastewater standardized in the Japanese Industrial Standards (JIS K 0102 46.3). Step 1 (preparation of a coloring agent): Hexaammonium heptamolybdate tetrahydrate (300 mg) and antimony potassium tartrate trihydrate (12 mg) were dissolved in deionized water (5 mL). Aqueous H_2_SO_4_ (77 wt %, 6 mL) was added to the solution and diluted with deionized water in a 25 mL measuring flask. An (+)-ʟ-ascorbic acid aqueous solution (7.2 w/v %, 5 mL) was added to the solution before the measurements. Step 2 (sample preparation): A potassium peroxodisulfate aqueous solution (4.0 w/v %, 5 mL) was added to the diluted phytic acid solution (25 mL), and the resulting mixture was autoclaved (121 °C, 30 min). Thereafter, the solution was diluted with deionized water to a concentration of approximately 1 µg P/mL. Step 3 (measurements): The total P concentration was measured via absorption spectrophotometry. The coloring agent (2 mL) was added to the diluted samples (25 mL), thoroughly mixed, and left for 15 min. After 15 min, the absorbance of each sample was measured at 880 nm using a V-650 spectrophotometer (JASCO, Tokyo, Japan). A standard curve was constructed using potassium dihydrogen phosphate solutions (0.5, 1.0, and 1.5 µg P/mL) as the standard solutions.

### Diaryl phosphate synthesis and characterization

The synthesis methods for diaryl phosphates using commercial and extracted phytic acid are included in Supporting Information File 1. The isolated compounds were characterized via NMR (^1^H, ^13^C, ^19^F, and ^31^P) and low-resolution mass spectrometry measurements. The NMR measurements were conducted using a Varian NMR 400 MHz system (400 MHz) and a Bruker AVANCE NEO console (400 MHz). Mass spectrometry was performed using LCMS-2020 (SHIMADZU, Kyoto, Japan) and JMS GC-mate II (JEOL, Tokyo, Japan) instruments. The melting points were determined using a Q-200 differential scanning calorimeter (TA Instruments, New Castle, DE, USA). The NMR spectra were recorded in a deuterated solvent (D_2_O, methanol-*d*_4_ or dimethyl sulfoxide (DMSO)-*d*_6_)), and the residual solvent was set to the chemical shift reference for ^1^H NMR (H_2_O, δ 4.79 ppm; MeOH, δ 3.31 ppm; DMSO, δ 2.50 ppm). All NMR spectra were compared with those reported in previous studies [15,46–48] and found to be consistent with the references. The spectra are provided in Supporting Information File 1.

## Supporting Information

File 1 Experimental section, characterization data and copies of spectra.

## Data Availability

Data generated and analyzed during this study is available from the corresponding author upon reasonable request.
