# Supplementary material for: Synthesis of diaryl phosphates using phytic acid as a phosphorus source
Source: Beilstein J Org Chem. 2026 Jan 30;22:213–23. doi: 10.3762/bjoc.22.15 (PMC12862610; doi:10.3762/bjoc.22.15)
Supplement: File 1 — Experimental section, characterization data and copies of spectra. [file Beilstein_J_Org_Chem-22-213-s001.pdf]

BEILSTEIN JOURNAL OF ORGANIC CHEMISTRY

Supporting Information

for

Synthesis of diaryl phosphates using phytic acid as a phosphorus source

Kazuya Asao, Seika Matsumoto, Haruka Mori, Riku Yoshimura, Takeshi Sasaki, Naoya Hirata, Yasuyuki Hayakawa and Shin-ichi Kawaguchi

Beilstein J. Org. Chem. **2026,** *22,* 213–223. doi:10.3762/bjoc.22.15

Experimental section, characterization data and copies of spectra

License and Terms: This is a supporting information file under the terms of the Creative Commons Attribution License (https://creativecommons.org/licenses/by/4.0). Please note that the reuse, redistribution and reproduction in particular requires that the author(s) and source are credited and that individual graphics may be subject to special legal provisions.
The license is subject to the *Beilstein Journal of Organic Chemistry* terms and conditions: (https://www.beilstein-journals.org/bjoc/terms)

1. Synthesis of diaryl phosphates from commercial phytic acid

1.1. Chemicals and analysis

All reagents were purchased from Nacalai Tesque (Kyoto, Japan), Tokyo Chemical Industry (Tokyo, Japan), FUJIFILM Wako Pure Chemical (Osaka, Japan), and Sigma-Aldrich (St. Louis, MO, USA). Phytic acid was purchased from Tokyo Chemical Industry. The Nuclear Magnetic Resonance (NMR) measurements were conducted using a Varian NMR 400 MHz system (400 MHz) and Bruker AVANCE NEO console (400 MHz). Mass spectrometry was performed using LCMS-2020 (SHIMADZU, Kyoto, Japan) and JMS GC-mate II (JEOL, Tokyo, Japan) instruments. The melting points were determined using a Q-200 differential scanning calorimeter (TA Instruments New Castle, DE, USA).

1.2. Synthesis method of diaryl phosphates using commercially available phytic acid

The phosphate ester synthesis was performed by using glass apparatus shown in Figure S1. All reactions were carried out with flowing N_2 (0.1 L/min) from a three-way cock for water removal. All diaryl phosphates were isolated as sodium salts.

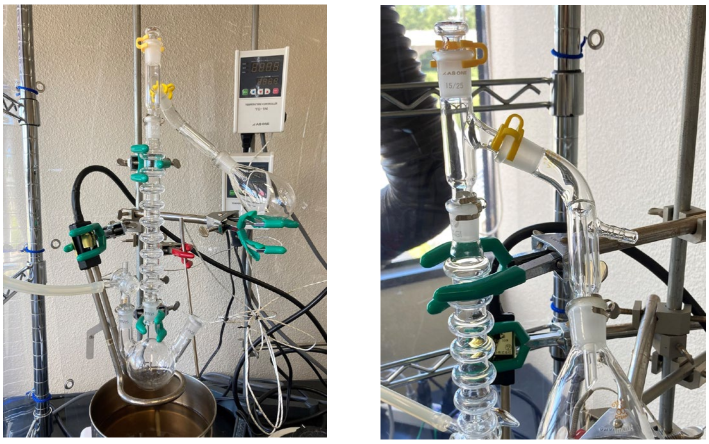

Figure S1. Glass apparatus used in the synthesis.

1.2.1. Synthesis of diaryl phosphates ***2a–j***

The reaction apparatus was set on the oil bath and N_2 flow was set to 0.1 L/min. Phytic acid (0.7 mmol, 50 wt % aqueous solution) was added to *N*,*N*-dimethylformamide (DMF, 6 mL). Triethylamine (12.6 mmol) and corresponding phenols (42 mmol) were poured into the mixture. The oil bath temperature was set to 200 °C and

the reaction was carried out for 48 hours under mixing with magnetic stirring. After the reaction was completed, the excess amount of phenol and DMF were removed by vacuum distillation by using a Kugelrohr apparatus set to 160 °C. The residue was diluted with toluene (30 mL) and washed with 1.2–2.0 M HCl (30 mL) twice. A few drops of 0.1% phenolphthalein (dissolved in 90% ethanol) were added to the collected organic layer, and the organic layer was neutralized with 1.0 M NaOH (methanol solution). The solution was evaporated, and the precipitate was filtered and washed with toluene. The residue was dried under vacuum to obtain the corresponding diaryl phosphate.

*1.2.2. Synthesis of dinaphthyl phosphates **2k, 2l***

The reaction apparatus was set on the oil bath and N_2 flow was set to 0.1 L/min. Phytic acid (0.7 mmol, 50 wt % aqueous solution) was added to DMF (6 mL). Triethylamine (12.6 mmol) and corresponding naphthols (42 mmol) were poured into the mixture. The oil bath temperature was set to 200 °C and the reaction was carried out for 48 hours under mixing with magnetic stirring. After the reaction was completed, the excess amount of phenol and DMF were removed by the vacuum distillation by using Kugelrohr apparatus set to 160 °C. The residue was diluted with 50 mL of toluene preheated at 60 °C and washed twice with 1.2 M HCl (50 mL, preheated at 60 °C). The organic layer was then washed with 50 mL of deionized water preheated at 60 °C. A few drops of 0.1% phenolphthalein (dissolved in 90% ethanol) were added to the collected organic layer, and the organic layer was neutralized with 1.0 M NaOH (methanol solution). The solution was evaporated, and the precipitate was filtered and washed with toluene. The residue was then washed with chloroform and dried under vacuum to obtain corresponding dinaphthyl phosphate.

1.2.3. Characterization of the isolated compounds

All of compounds are known, and the NMR spectral data were consistent with previously reported data for the corresponding free acid form or sodium salts [1-4].

Sodium diphenylphosphate (**2a**) (lit. [1,2], data for both form): light brown solid; 618 mg (2.27 mmol, 54%); ^{1}H NMR (400 MHz, D_2O): δ 7.39 (t, 4H, J = 8.0 Hz), 7.23–7.19 (m, 6H) ppm; ^{13}C NMR (100 MHz, D_2O): δ 151.7 (d, $J_{C–P}$ = 7.2 Hz), 129.8, 124.5, 120.2 (d, $J_{C–P}$ = 4.4 Hz) ppm; ^{31}P NMR (162 MHz, D_2O): δ −8.90 ppm; LRMS (ESI) $[M–Na]^-$ m/z = 249; melting point (m.p.): 214–215 °C.

Sodium bis(4-methylphenyl)phosphate (**2b**) (lit. [1], data for free acid form): brown solid; 707 mg (2.36 mmol, 56%); ^{1}H NMR (400 MHz, D_2O): δ 7.16 (d, 4H, J = 8.4 Hz), 7.07 (d, 4H, J = 8.0 Hz), 2.26 (s, 6H) ppm; ^{13}C NMR (100 MHz, D_2O): δ 149.4 (d, $J_{C–P}$ = 7.3 Hz), 134.3, 130.1, 119.9 (d, $J_{C–P}$ = 4.4 Hz), 19.8 ppm; ^{31}P NMR

(162 MHz, D_2O): δ −8.64 ppm; LRMS (ESI) $[M–Na]^-$ m/z = 277; m.p.: 230–233 °C.

Sodium bis(3,5-dimethylphenyl)phosphate **2d** (lit. [1], data for free acid form): brown solid; 934 mg (2.84 mmol, 63%); ^{1}H NMR (400 MHz, D_2O): δ 6.72 (s, 4H), 6.55 (s, 2H), 2.05 (s, 12H) ppm ; ^{13}C NMR (100 MHz, D_2O): δ 154.3 (d, $J_{C–P}$ = 7.4 Hz), 142.0, 127.9, 120.1 (d, $J_{C–P}$ = 4.7 Hz), 22.9 ppm; ^{31}P NMR (162 MHz, D_2O): δ −7.04 ppm; LRMS (FAB) $[M+H]^+$ m/z = 329, $[M–Na+2H]^+$ m/z = 307; m.p.: 239–241 °C.

Sodium bis(4-*tert*-butylphenyl)phosphate (**2e**) (lit. [1,2], data for both form): brown solid; 905 mg (2.35 mmol, 56%); ^{1}H NMR (400 MHz, CD_3OD): δ 7.30 (br s, 4H), 7.14 (br s, 4H), 1.29 (s, 18H) ppm; ^{13}C NMR (100 MHz, CD_3OD): δ 150.6 (d, $J_{C–P}$ = 7.1 Hz), 145.8, 125.6, 119.4 (d, $J_{C–P}$ = 4.8 Hz), 33.7, 30.6 ppm; ^{31}P NMR (162 MHz, CD_3OD): δ −9.66 ppm; LRMS (ESI) $[M–Na]^-$ m/z = 361; m.p.: not observed (decomposed at approximately 350 °C).

Sodium bis(4-fluorophenyl)phosphate (**2f**) (lit. [1], data for free acid form): brown solid; 601 mg (1.95 mmol, 46%); ^{1}H NMR (400 MHz, D_2O): δ 7.25–7.15 (m, 4H), 7.15–7.05 (m, 4H) ppm; ^{13}C NMR (100 MHz, D_2O): δ 159.1 (d, $J_{C–F}$ = 238 Hz), 147.6 (dd, $J_{C–F}$ = 2.1 Hz, J_{C-P} = 7.2 Hz), 121.6 (dd, $J_{C–F}$ = 8.5 Hz, $J_{C–P}$ = 4.4 Hz), 116.1 (d, $J_{C–F}$ = 23.5 Hz) ppm; ^{19}F NMR (376 MHz, D_2O): δ −119.6 ppm; ^{31}P NMR (162 MHz, D_2O): δ −8.59 ppm; LRMS (ESI) $[M–Na]^-$ m/z = 285; m.p.: 177–184 °C.

Sodium bis(4-chlorophenyl)phosphate (**2g**) (lit. [2], data for sodium salts): brown solid; 859 mg (2.52 mmol, 60%) ^{1}H NMR (400 MHz, D_2O): δ 7.25 (d, 4H, J = 8.4 Hz), 7.09 (d, 4H, J = 8.4 Hz) ppm; ^{13}C NMR (100 MHz, D_2O): δ 150.3 (d, $J_{C–P}$ = 7.1 Hz), 129.5, 128.9, 121.5 (d, $J_{C–P}$ = 4.6 Hz) ppm; ^{31}P NMR (162 MHz, D_2O): δ −9.59 ppm; LRMS (ESI) $[M–Na]^-$ m/z = 317; m.p.: 232–236 °C.

Sodium bis(4-methoxyphenyl)phosphate (**2h**) (lit. [2], data for sodium salts): brown solid; 832 mg (2.51 mmol, 60%); ^{1}H NMR (400 MHz, D_2O): δ 7.11 (d, 4H, J = 8.4 Hz), 6.90 (d, 4H, J = 8.8 Hz), 3.76 (s, 6H) ppm; ^{13}C NMR (100 MHz, D_2O): δ 155.3, 145.7 (d, $J_{C–P}$ = 7.3 Hz), 121.2 (d, $J_{C–P}$ = 4.3 Hz), 114.8, 55.7 ppm; ^{31}P NMR (162 MHz, D_2O): δ −8.18 ppm; LRMS (ESI) $[M–Na]^-$ m/z = 309; m.p.: 200–205 °C.

Sodium bis(naphthalen-1-yl)phosphate (**2k**) (lit. [3], data for free acid form): brown solid; 399 mg (1.07 mmol, 25%); ^{1}H NMR (400 MHz, DMSO-d_6): δ 8.13 (d, 2H, J = 8.4 Hz), 7.84 (d, 2H, J = 7.2 Hz), 7.57 (d, 2H, J = 7.6 Hz), 7.52 (d, 2H, J = 8.0 Hz), 7.48–7.40 (m, 4H), 7.37 (t, 2H, J = 8.0 Hz) ppm; ^{13}C NMR (100 MHz, DMSO-d_6): δ 149.6 (d, $J_{C–P}$ = 7.0 Hz), 134.1, 127.3, 126.8 (d, $J_{C–P}$ = 6.6 Hz), 125.9 (2C), 125.1, 122.4, 121.4, 114.1 (d, $J_{C–P}$ = 1.6 Hz) ppm; ^{31}P NMR (162 MHz, DMSO-d_6): δ −10.6 ppm; LRMS (ESI) $[M–Na]^-$ m/z = 349; m.p.: 262–271 °C.

Sodium bis(naphthalen-2-yl)phosphate (**2l**) (lit. [4], data for free acid form): light yellow solid; 14.1 mg (0.0379 mmol, 1%); ^{1}H NMR (400 MHz, DMSO-d6): δ 7.83–7.74 (m, 6H), 7.67 (s, 2H), 7.45–7.41 (m, 2H), 7.38–7.33 (m, 4H) ppm (lit. [4]); ^{13}C NMR (100 MHz, DMSO-d6): δ 151.7 (d, $J_{C–P}$ = 7.0 Hz), 133.9, 129.2, 128.5, 127.4, 126.9, 126.0, 124.0, 121.5 (d, $J_{C–P}$ = 5.5 Hz), 115.1 (d, $J_{C–P}$ = 4.7 Hz) ppm (lit. [4]); ^{31}P NMR (162 MHz, DMSO-d6): δ −11.1 ppm; LRMS (ESI) $[M–Na]^-$ m/z = 349.

1.3. NMR spectra for synthesized compounds

*1.3.1. Sodium diphenylphosphate (**2a**)*

^{1}H NMR (400 MHz, D_2O).

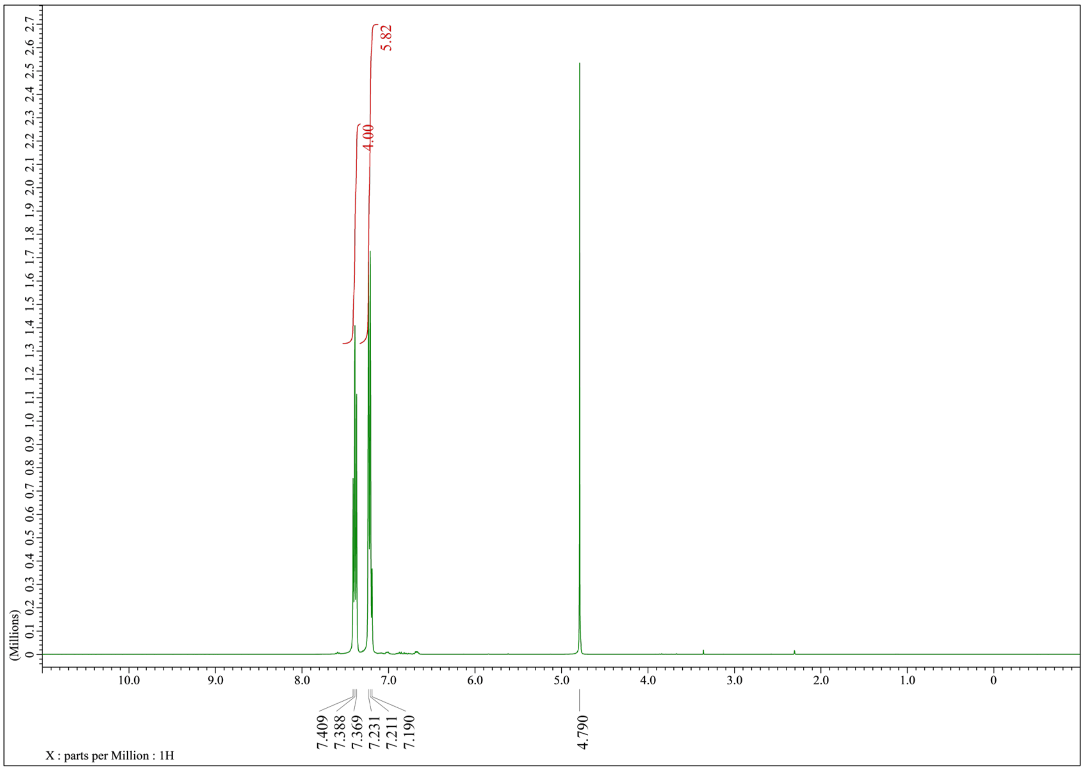

^{13}C NMR (100 MHz, D_2O).

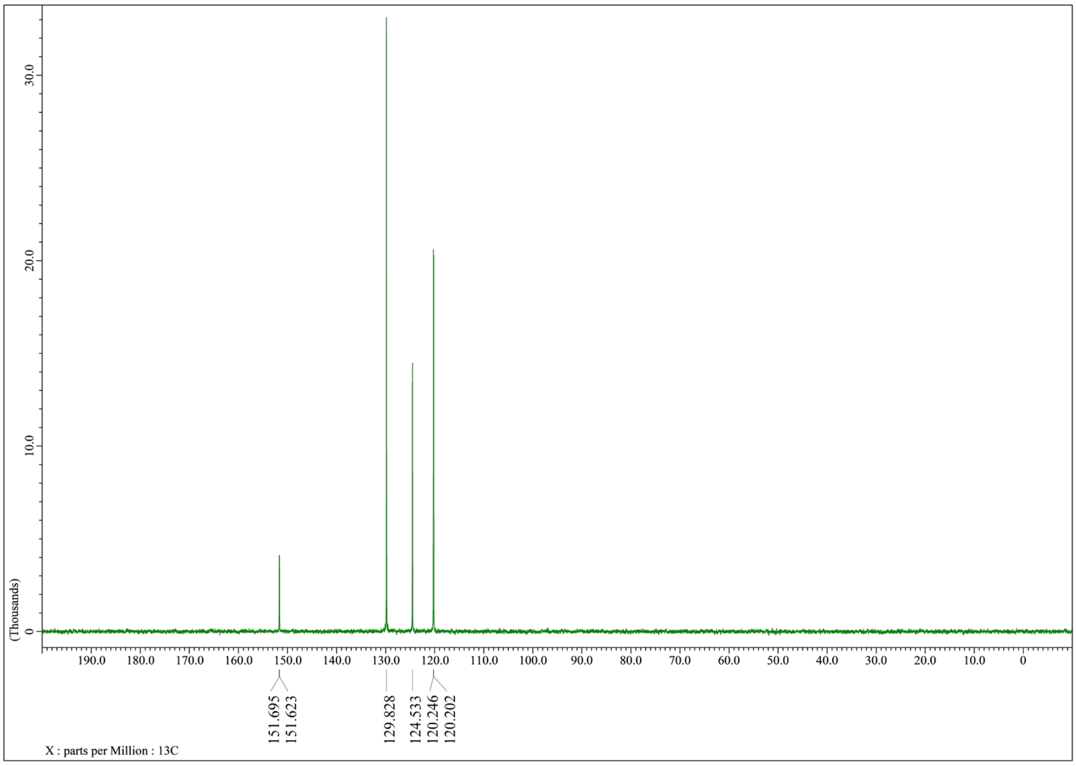

^{31}P NMR (162 MHz, D_2O).

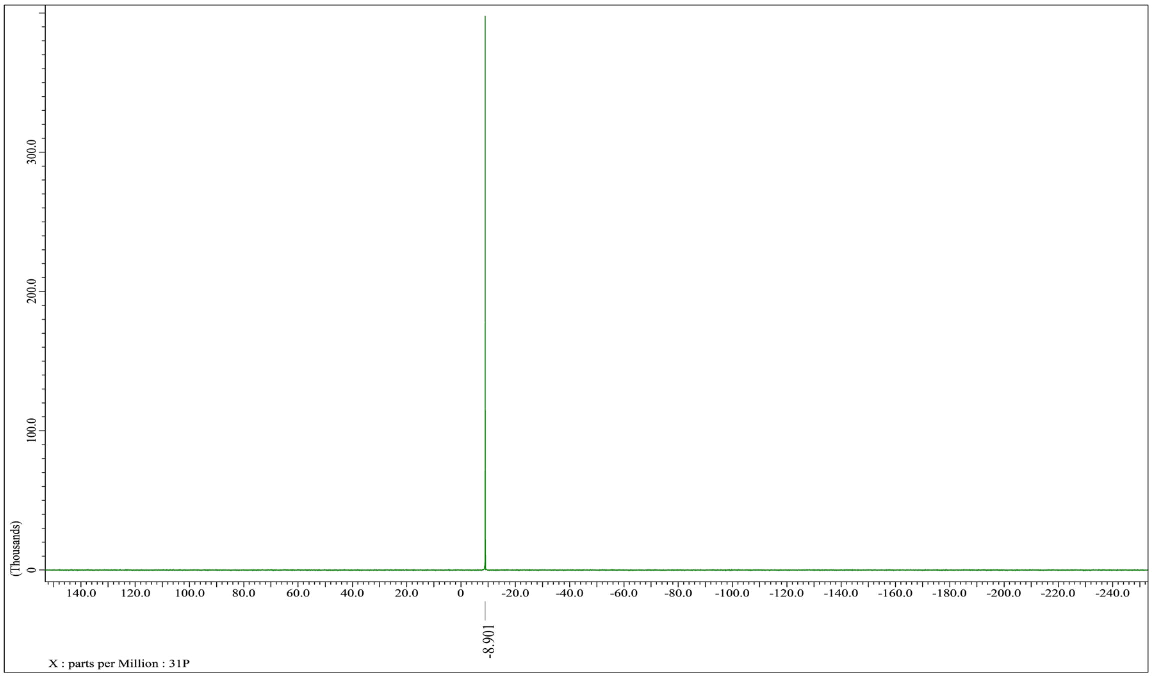

*1.3.2. Sodium bis(4-methylphenyl)phosphate (**2b**)*

^{1}H NMR (400 MHz, D_2O).

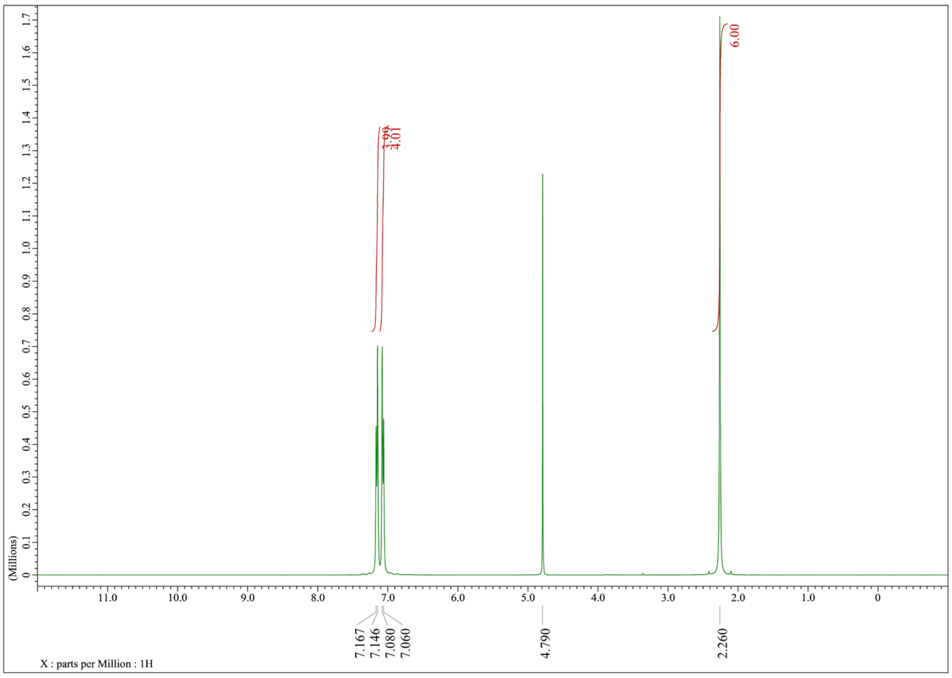

^{13}C NMR (100 MHz, D_2O).

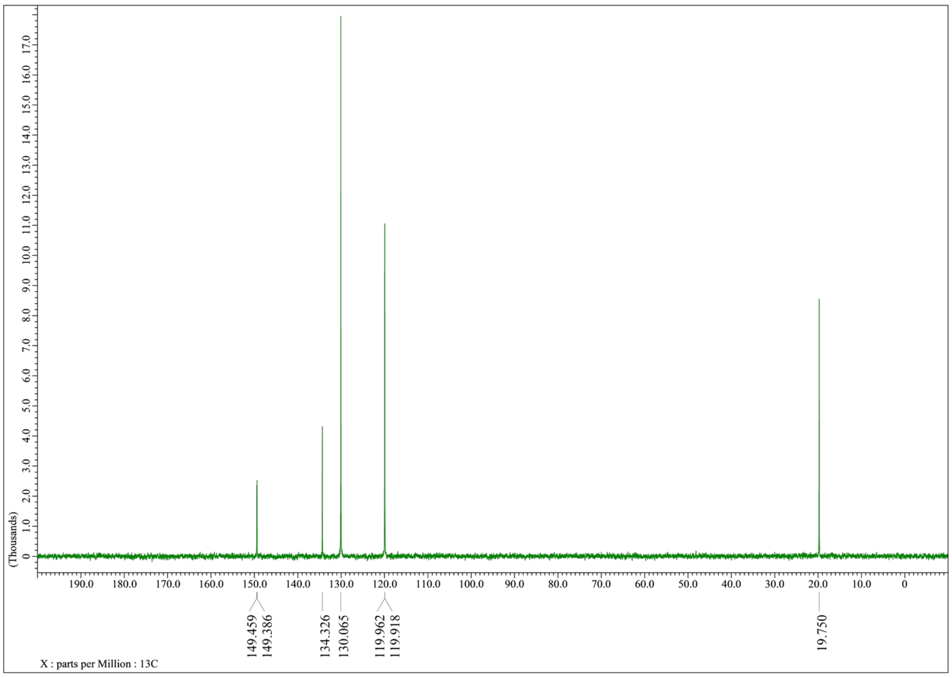

^{31}P NMR (162 MHz, D_2O).

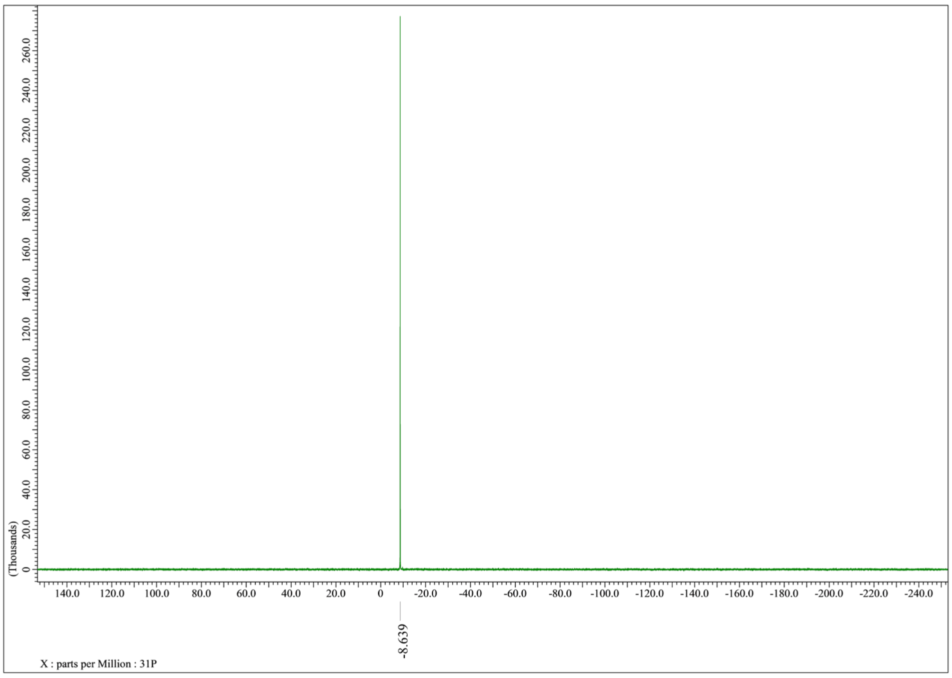

*1.3.3. Sodium bis(3,5-dimethylphenyl)phosphate (**2d**)*

^{1}H NMR (400 MHz, D_2O).

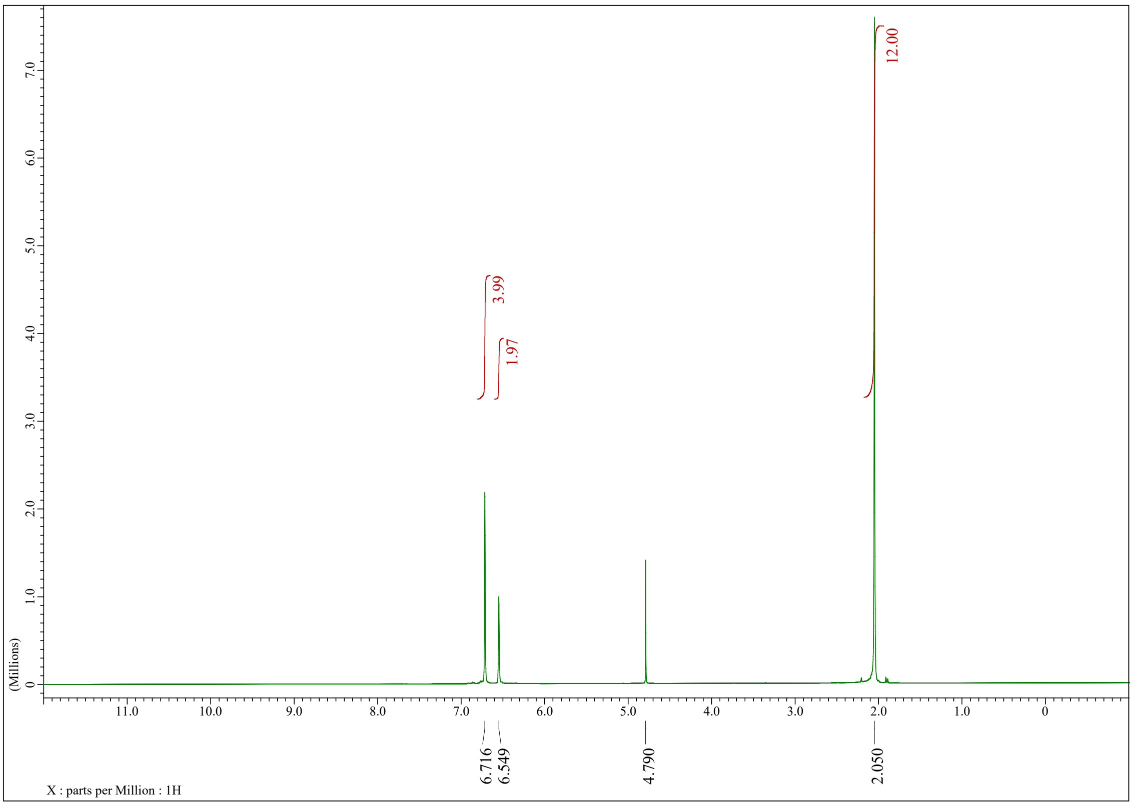

^{13}C NMR (100 MHz, D_2O).

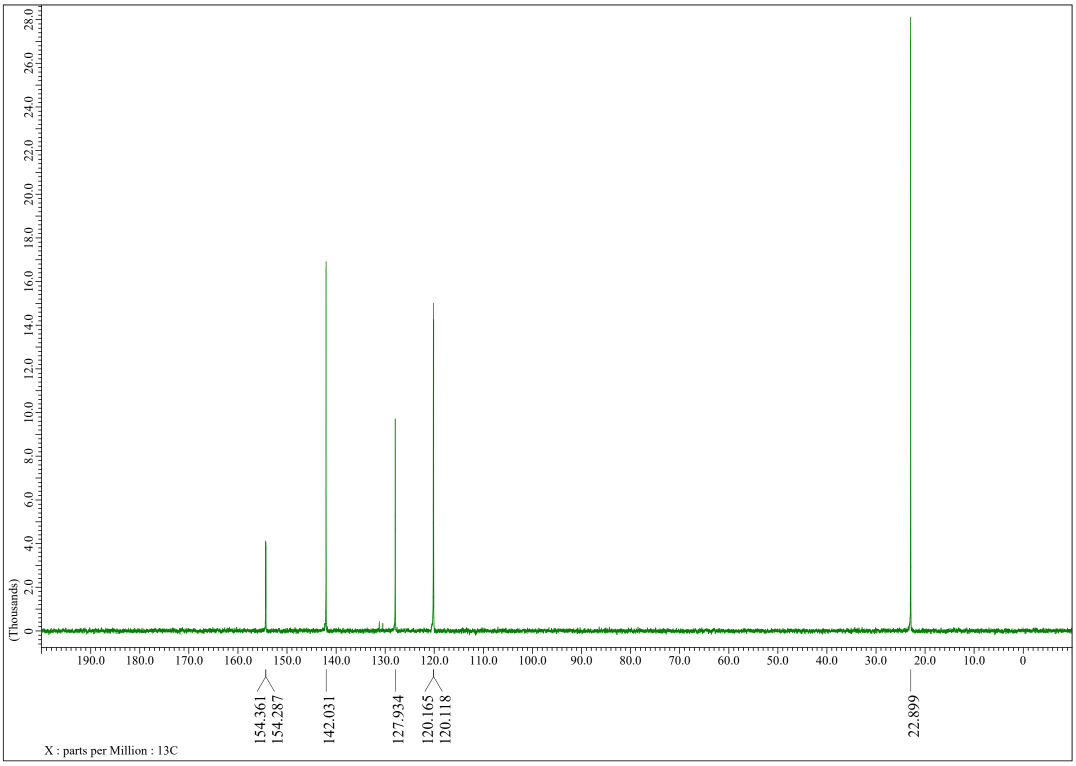

^{31}P NMR (162 MHz, D_2O).

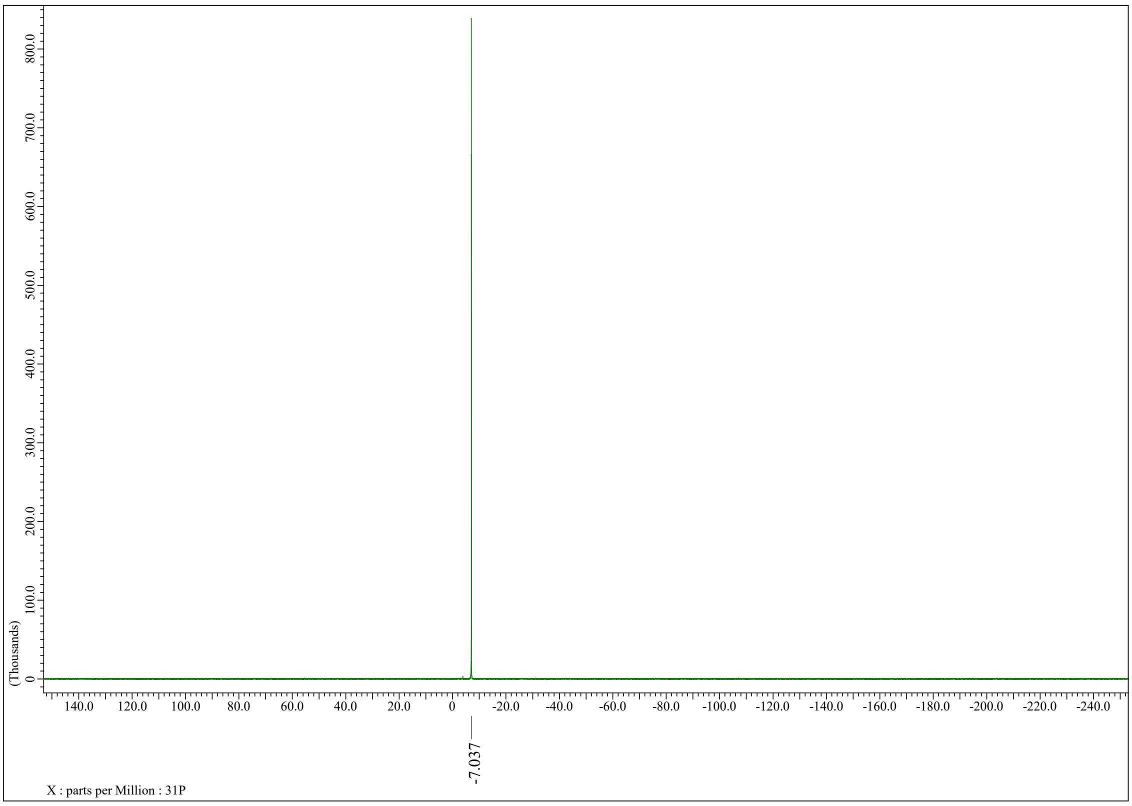

*1.3.4. Sodium bis(4-tert-butylphenyl)phosphate (**2e**)*

^{1}H NMR (400 MHz, CD_3OD).

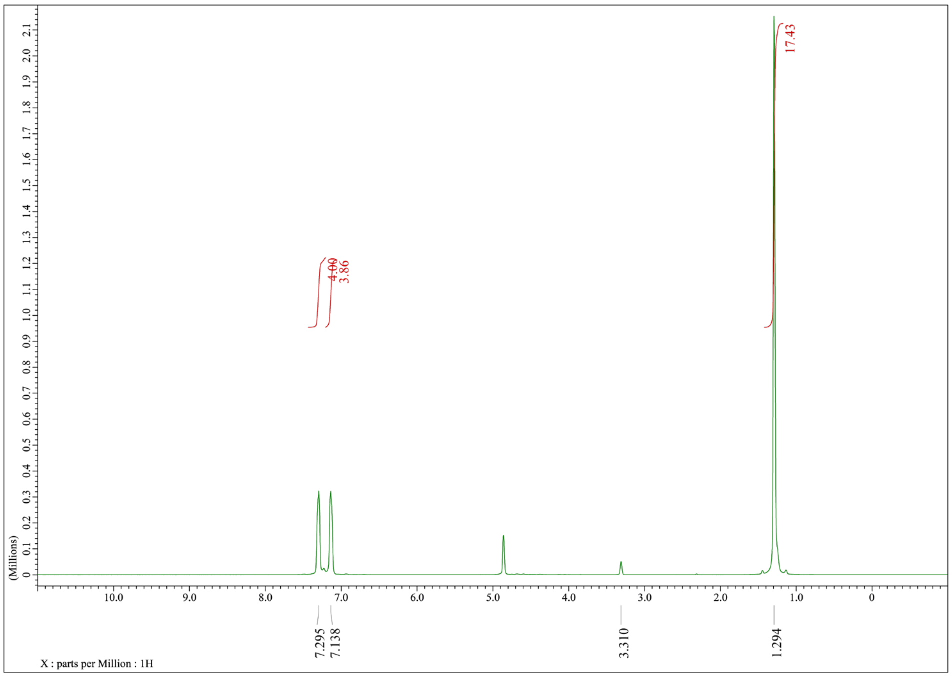

^{13}C NMR (400 MHz, CD_3OD).

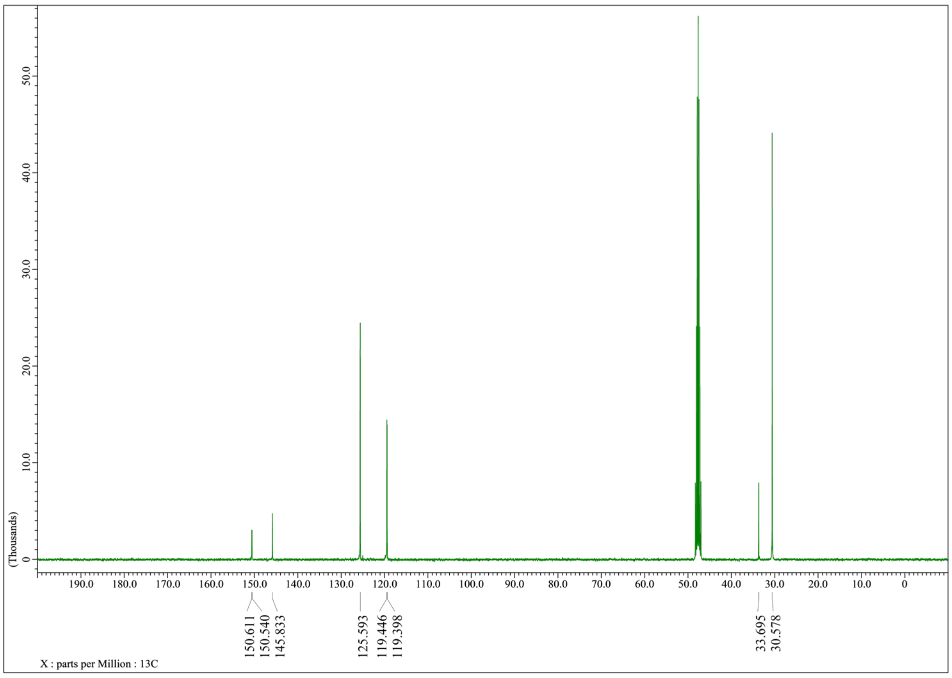

^{31}P NMR (162 MHz, CD_3OD).

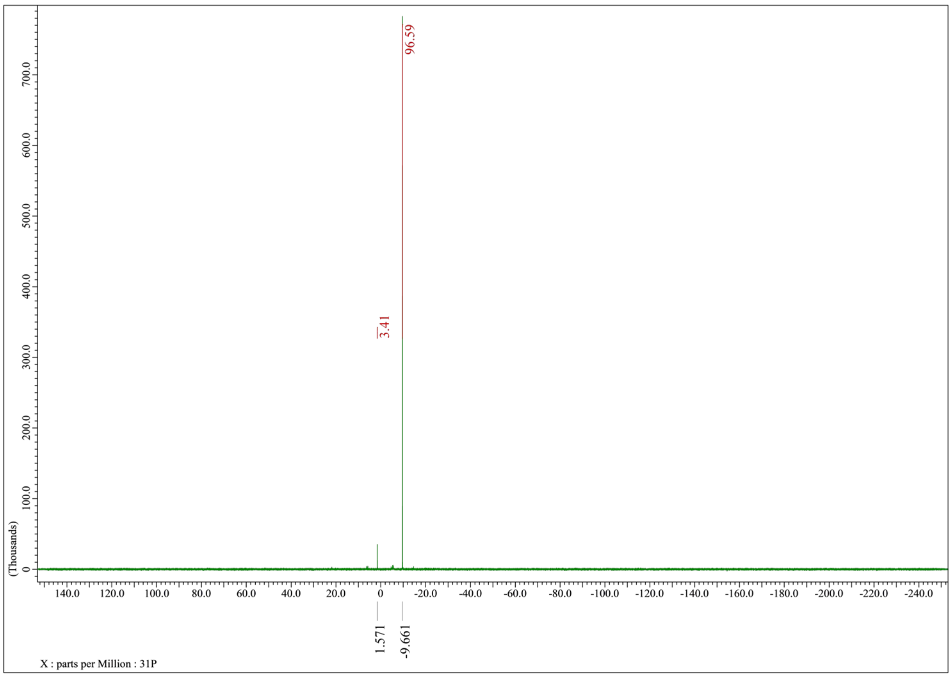

*Contains 3.41 mol % of sodium phosphate as an impurity.

*1.3.5. Sodium bis(4-fluorophenyl)phosphate (**2f**)*

^{1}H NMR (400 MHz, D_2O).

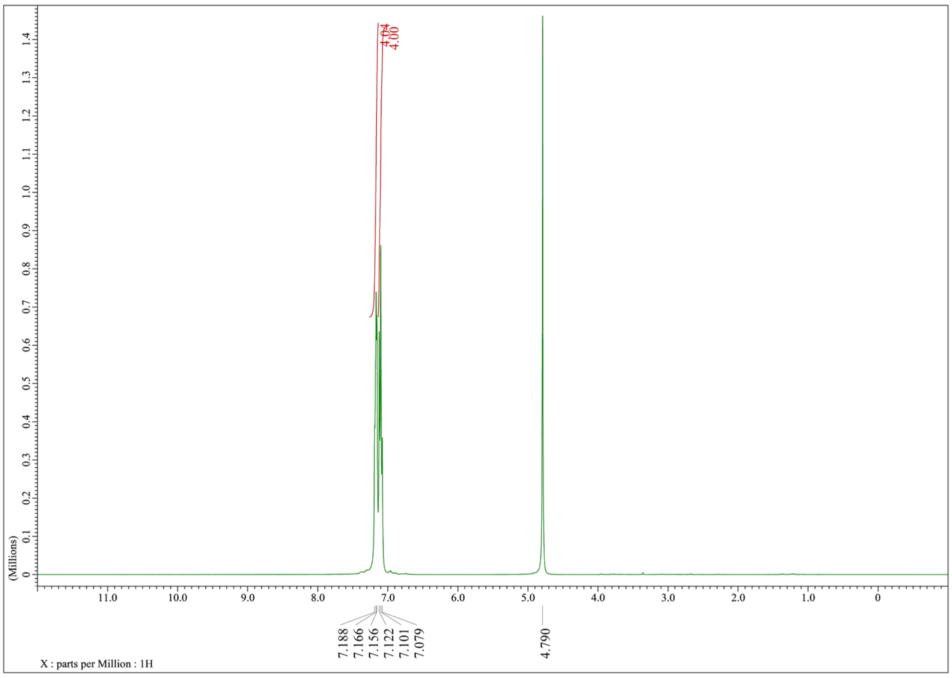

^{13}C NMR (100 MHz, D_2O).

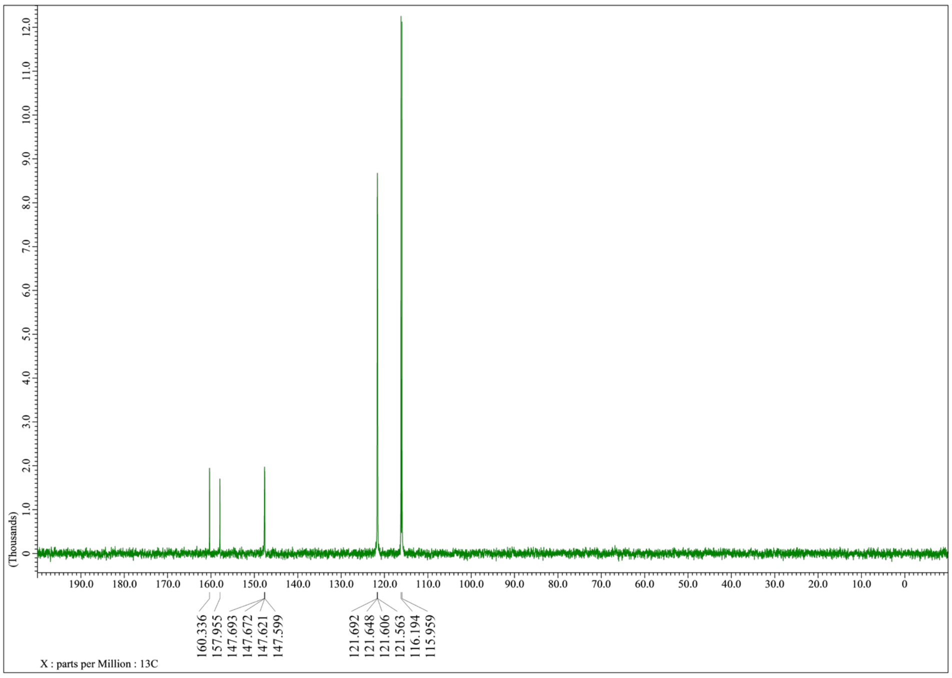

^{19}F NMR (376 MHz, D_2O).

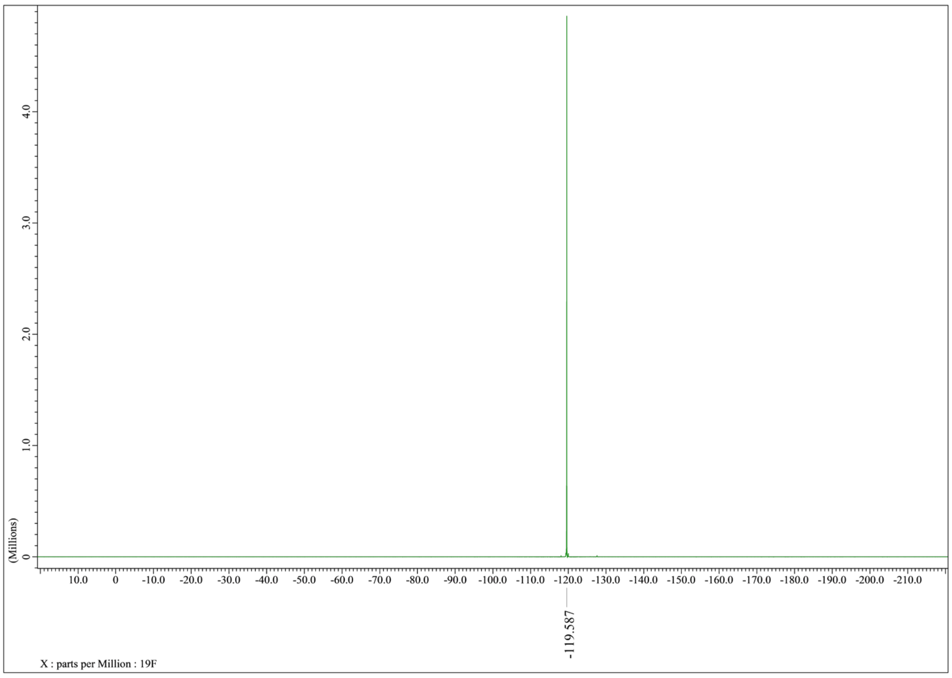

^{31}P NMR (162 MHz, D_2O).

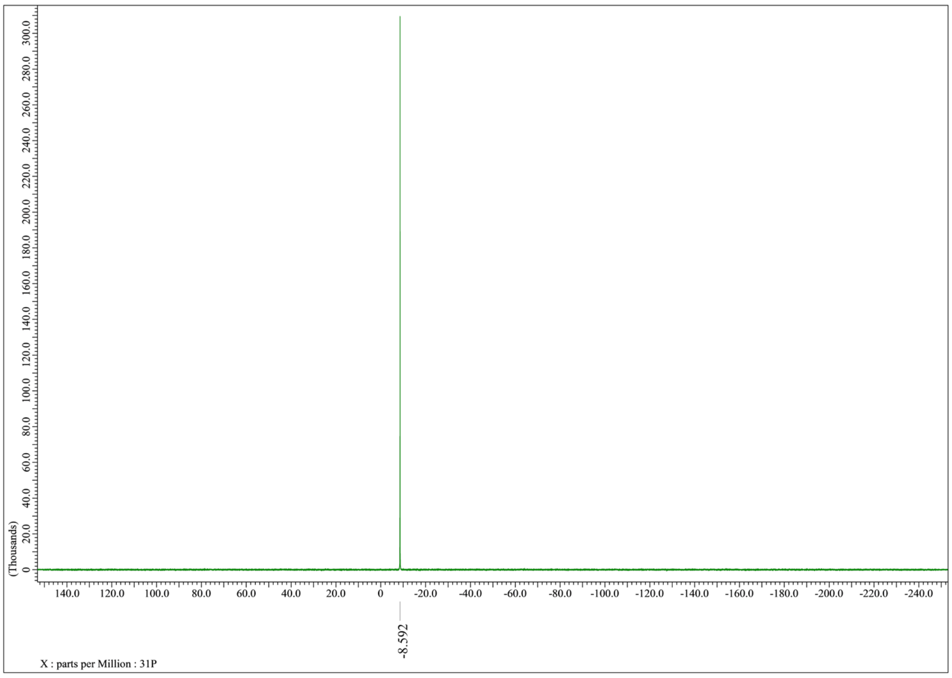

*1.3.6. Sodium bis(4-chlorophenyl)phosphate (**2g**)*

^{1}H NMR (400 MHz, D_2O).

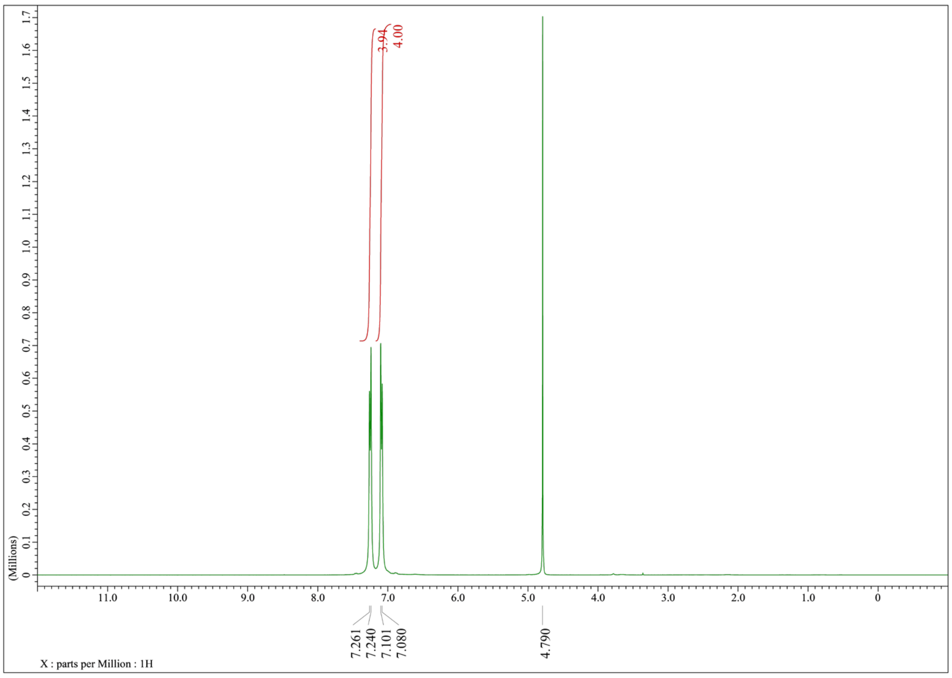

^{13}C NMR (100 MHz, D_2O).

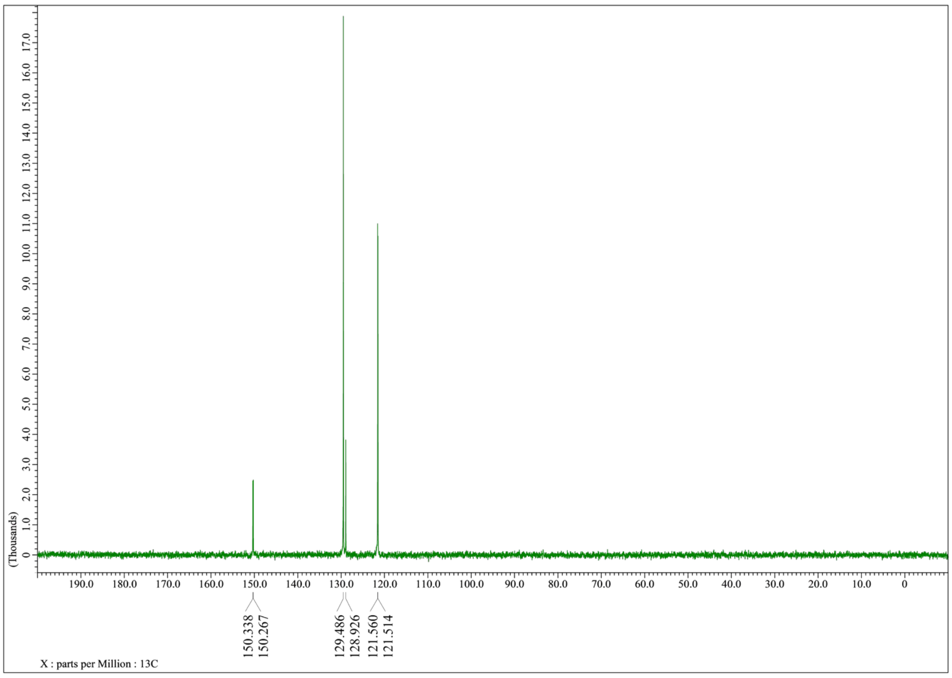

^{31}P NMR (162 MHz, D_2O).

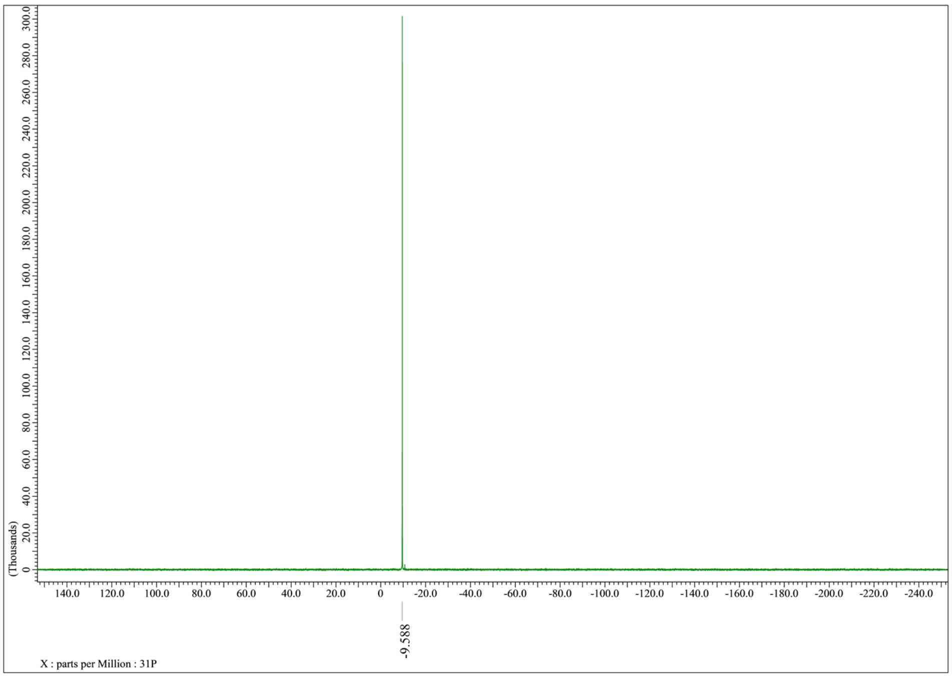

*1.3.7. Sodium bis(4-methoxyphenyl)phosphate (**2h**)*

^{1}H NMR (400 MHz, D_2O).

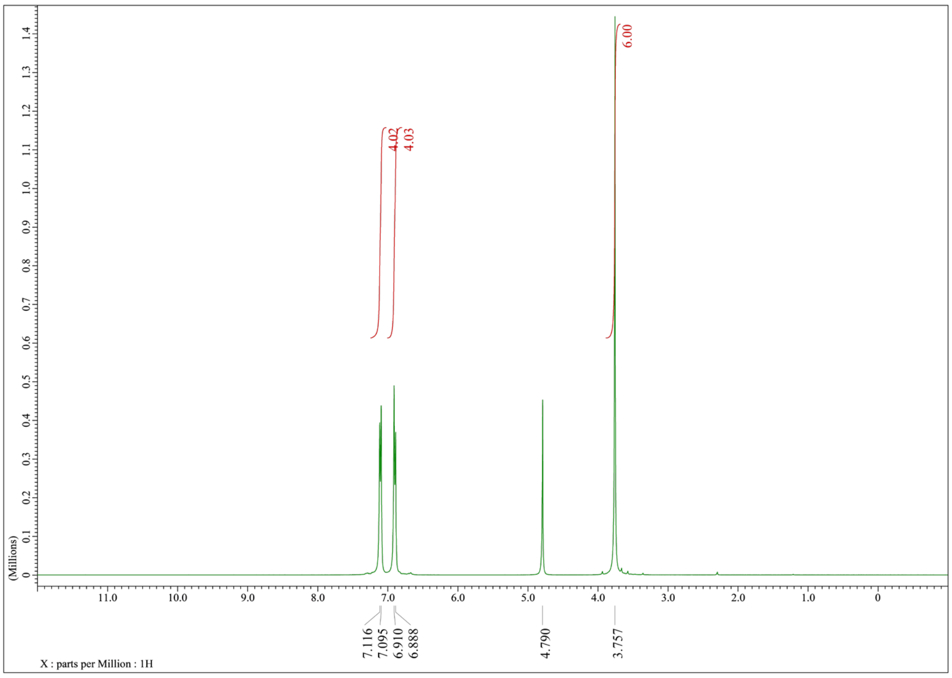

^{13}C NMR (100 MHz, D_2O).

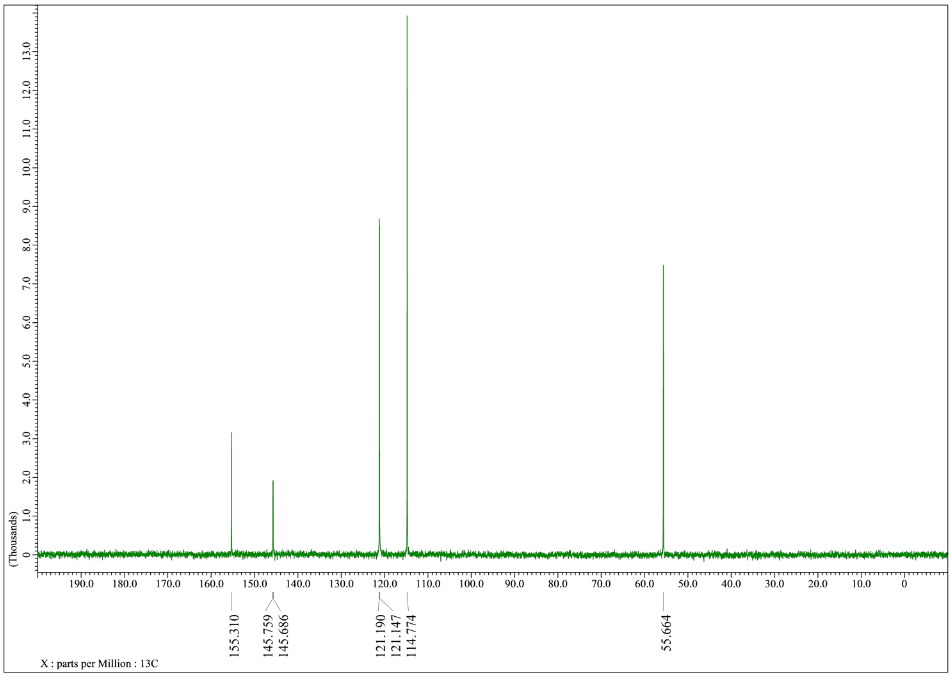

^{31}P NMR (162 MHz, D_2O).

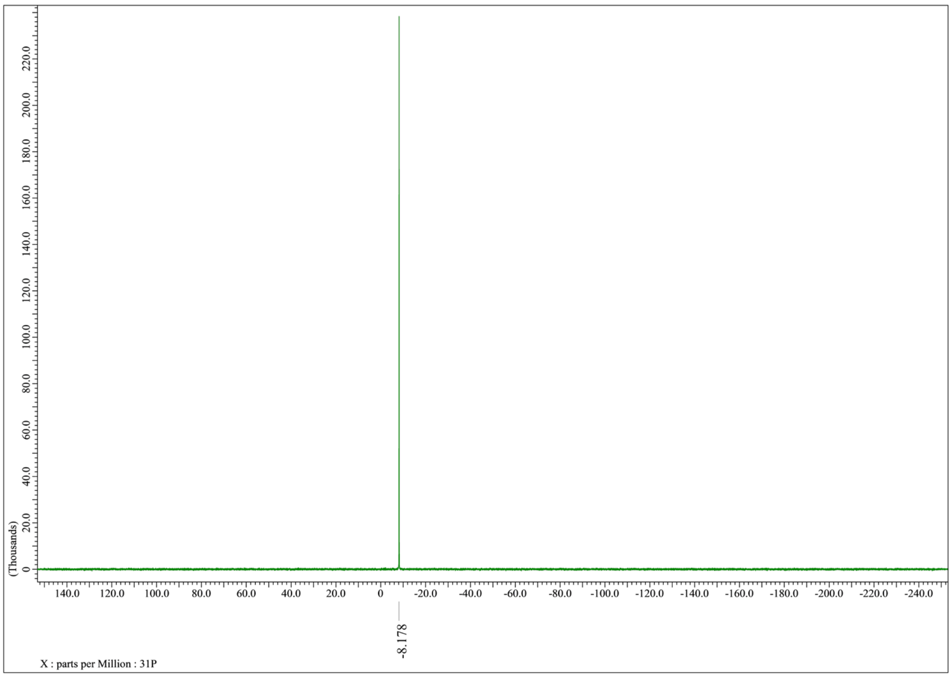

*1.3.8. Sodium bis(naphthalen-1-yl) phosphate (**2k**)*

^{1}H NMR (400 MHz, DMSO-d_6)

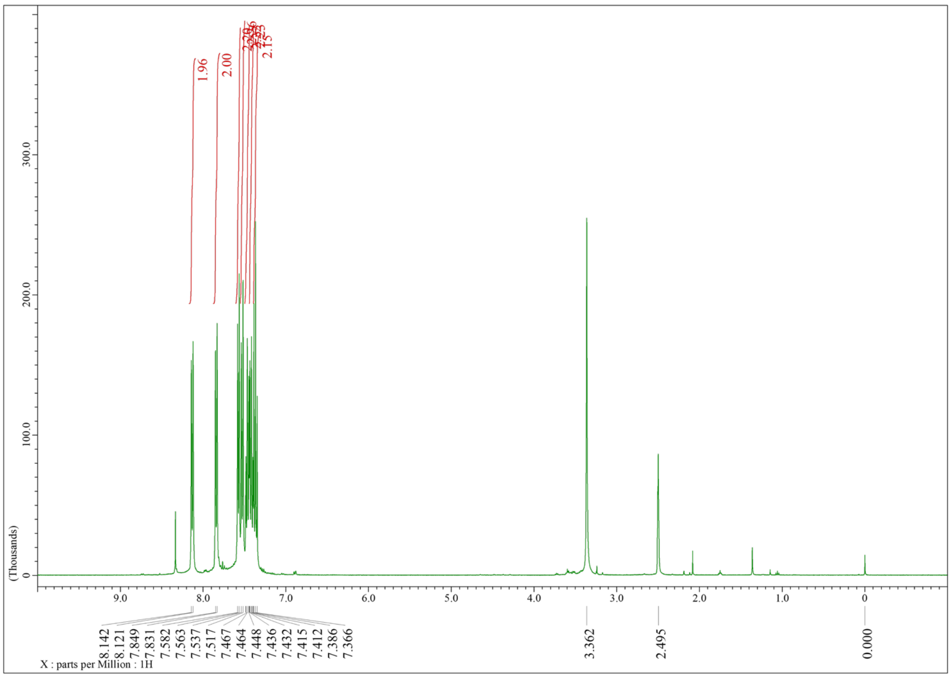

^{13}C NMR (100 MHz, DMSO-d_6).

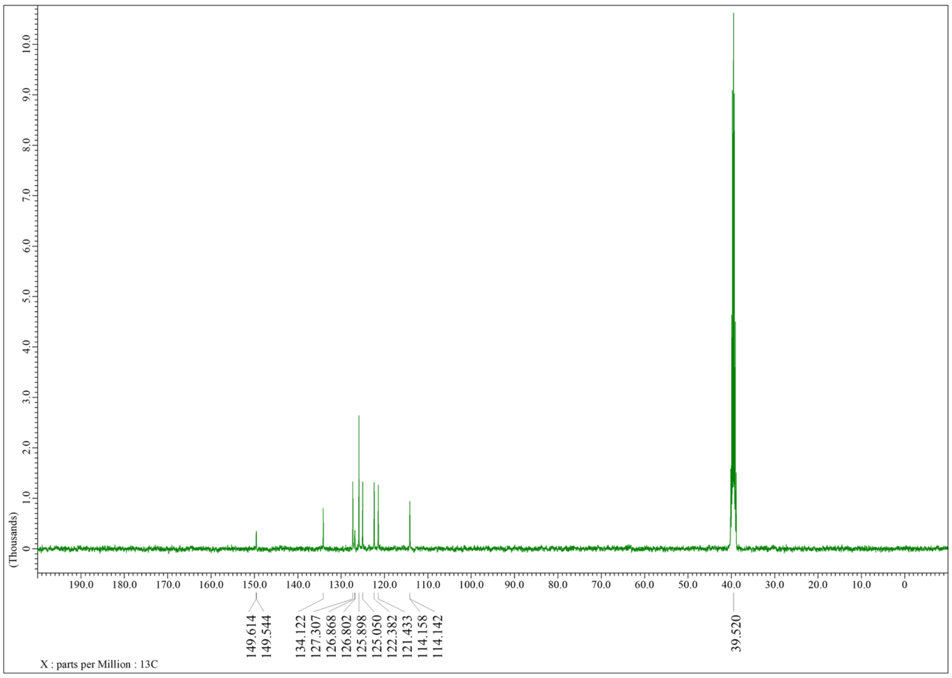

^{31}P NMR (162 MHz, DMSO-d_6).

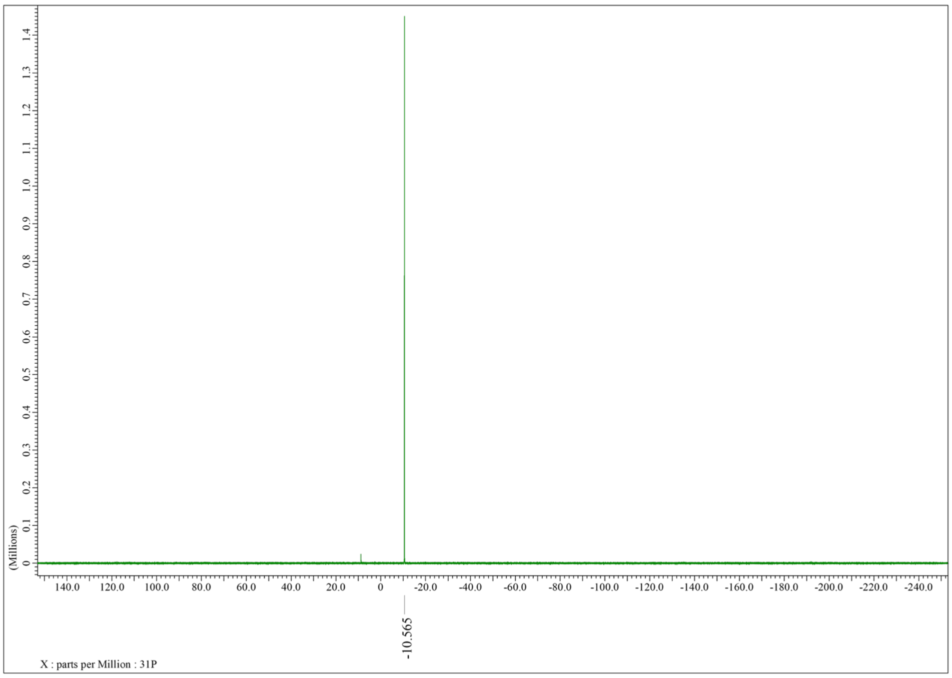

*1.3.9. Sodium bis(naphthalen-2-yl) phosphate (**2l**)*

^{1}H NMR (400 MHz, DMSO-d_6)

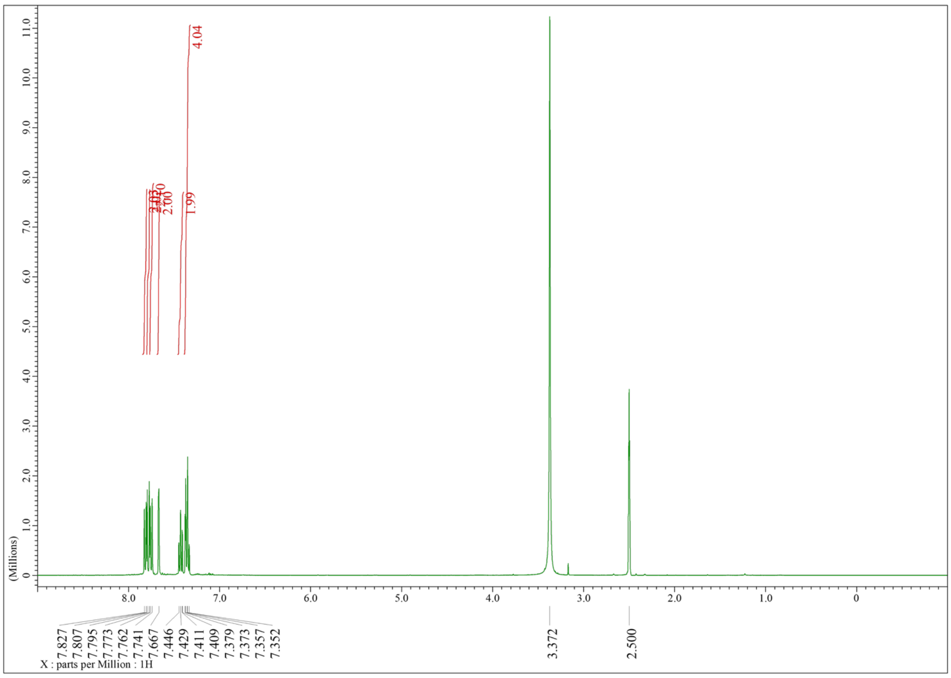

^{13}C NMR (100 MHz, DMSO-d_6).

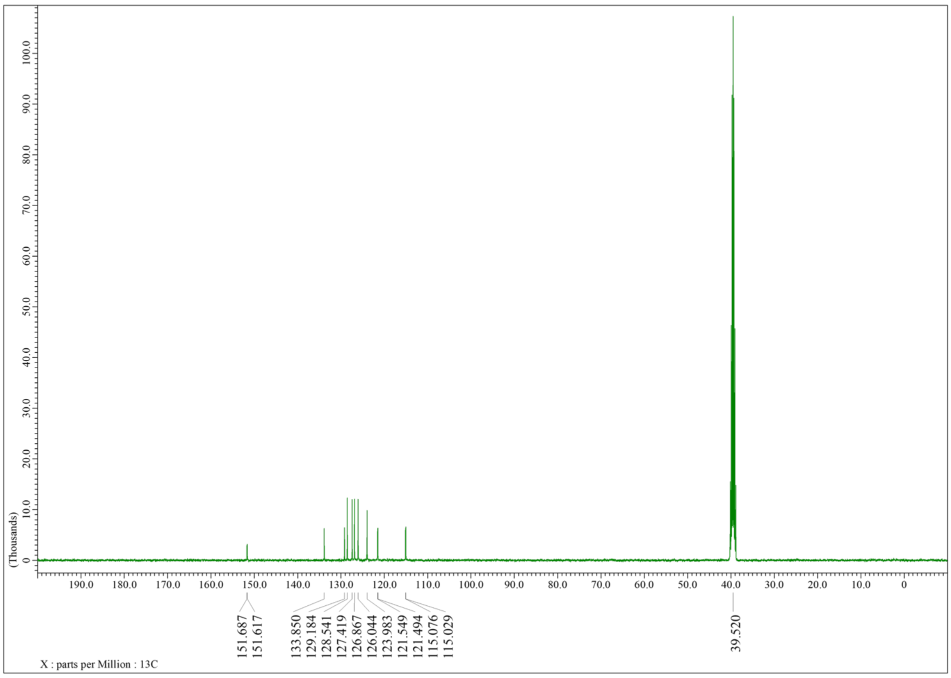

^{31}P NMR (162 MHz, DMSO-d_6).

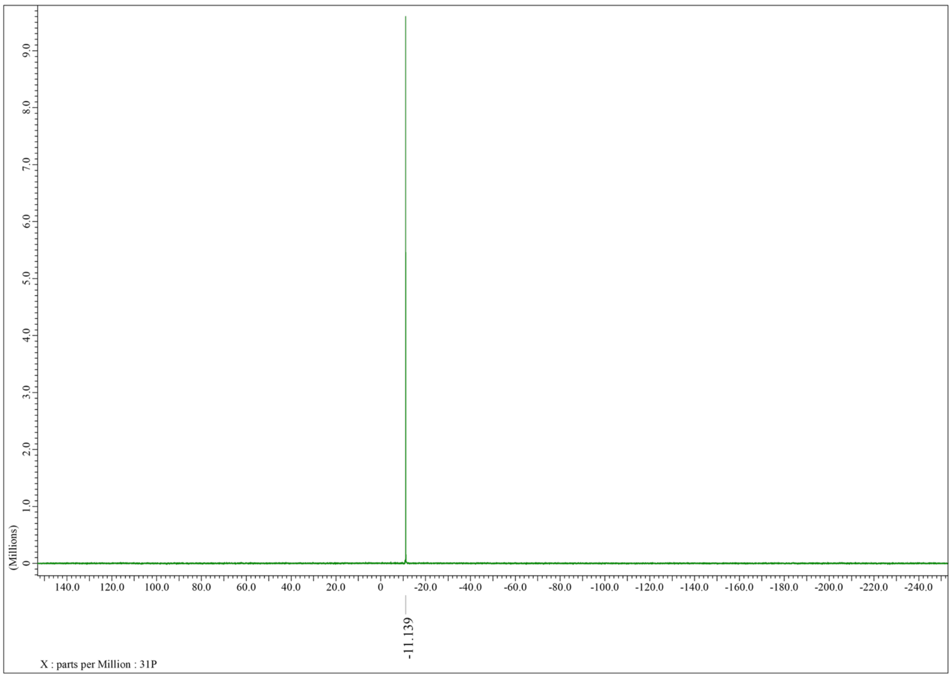

1.4. Synthesis of diaryl phosphate standards for reaction yield determination.

*1.4.1. Synthesis of bis(2,6-dimethylphenyl)phosphate (**2c**) using phosphoric acid*

The standard **2c** was prepared referring to Tran et al. [1] The sodium salt of **2c** was highly hygroscopic; therefore, **2c** was isolated in its free acid form.

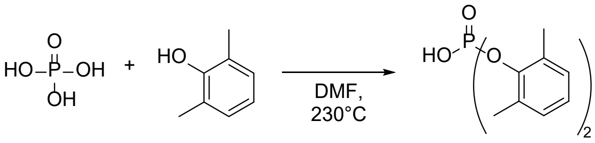

1c **2c**

The reaction apparatus was set on the oil bath and N_2 flow was set to 0.1 L/min. Phosphoric acid (4.03 mmol, 85 wt % aqueous solution) was added to DMF (6 mL) and 2,6-xylenol (40.8 mmol). The oil bath temperature was set to 230 °C and the reaction was carried out for 48 hours under mixing with magnetic stirrer. After the reaction was completed, the reaction mixture was dissolved in 1 mL of DMF. The mixture was then diluted with toluene (30 mL) and washed with 1.2 M HCl (30 mL) twice. A few drops of 0.1% phenolphthalein (dissolved in 90% ethanol) were added to the collected organic layer, and the organic layer was neutralized with NaOH (1.0 M methanol solution). The solution was evaporated, and the precipitate was filtered and washed with toluene. The residue was dissolved in deionized water (27 mL). Chloroform (30 mL) and conc. HCl (3 mL) were added to the solution. The solution was well-shaken, and the organic layer was collected. The organic layer was washed again with 1.2 M HCl (30 mL). The organic layer was then dried with Na_2SO_4, and the solvent was evaporated under reduced pressure. The residue was dried in vacuo to obtain **2c** as a pink solid; 614 mg (2.01 mmol, 50%); ^{1}H NMR (400 MHz, $CDCl_3$): δ 11.70 (s, 1H), 6.96 (s, 6H), 2.18 (s, 12H) ppm; ^{13}C NMR (100 MHz, $CDCl_3$): δ 148.1 (d, $J_{C\text{–}P}$ = 8.3 Hz), 130.3 (d, $J_{C\text{–}P}$ = 3.2 Hz), 128.9 (d, $J_{C\text{–}P}$ = 1.4 Hz), 125.2 (d, $J_{C\text{–}P}$ = 1.7 Hz), 16.8 ppm; ^{31}P NMR (162 MHz, $CDCl_3$): δ -8.11 ppm; LRMS (ESI) $[M\text{–}H]^-$ m/z = 305; m.p.: 134–136 °C.

The spectral data were consistent with previously reported data [1].

*1.4.2. Synthesis of bis(4-cyanophenyl)phosphate (**2j**) using $POCl_3$*

The standard was prepared using phosphoryl oxychloride.

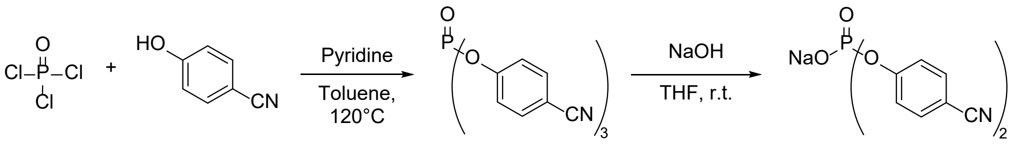

1j **3j** **2j**

Solid **1j** (1786 mg, 15.00 mmol), dehydrated toluene (20 mL), and dehydrated pyridine (709 mg, 8.97 mmol) were added to a well-dried 50 mL two-neck round-bottomed flask and stirred using a magnetic stirrer. After the mixture was completely dissolved, $POCl_3$ (462 mg, 3.01 mmol) was added dropwise. The reaction was then carried out at 120 °C for 12 hours. After the reaction was completed, the mixture was diluted with toluene (80 mL) and quenched with 0.5 M Na_2CO_3 (100 mL). The organic layer was separated, and the remaining aqueous layer was further extracted with toluene (100 mL) twice. The collected organic layer was dried with Na_2SO_4, and the solvent was evaporated under reduced pressure. The residue was dried in vacuo to obtain a crude mixture of **1j** and **3j** (543 mg). The purity of **3j** was 89.15 wt % according to ^{1}H NMR, and the crude was used for a subsequent reaction without further purification.

The crude (317 mg, 0.704 mmol of **3j**) and tetrahydrofuran (THF, 10 mL) were added into a 50 mL two-neck round-bottomed flask, and NaOH (1.0 M MeOH solution, 0.704 mmol) was added to the mixture. The mixture was then stirred using a magnetic stirrer overnight at room temperature. After the reaction, the mixture was diluted with 30 mL of toluene, and the solvent was evaporated under reduced pressure. The residue was dispersed in THF and toluene, and the solid was filtered and dried in vacuo to obtain **2j** as white needles; 209 mg (0.645 mmol, 37%, 2 steps); ^{1}H NMR (400 MHz, D_2O): δ 7.76 (d, 4H, J = 8.8 Hz), 7.32 (d, 4H, J = 8.8 Hz) ppm; ^{13}C NMR (100 MHz, D_2O): δ 155.3 (d, $J_{C–P}$ = 7.0 Hz), 134.5, 121.0 (d, $J_{C–P}$ = 5.0 Hz), 119.4, 106.9 ppm; ^{31}P NMR (162 MHz, D_2O): δ -10.95 ppm; LRMS (ESI) $[M–Na]^-$ m/z = 299; m.p.: 282–286 °C.

The spectral data were consistent with previously reported data [2].

*1.4.3. NMR spectra for synthesized standards: bis(2,6-dimethylphenyl)phosphate (**2c**)*

^{1}H NMR (100 MHz, $CDCl_3$).

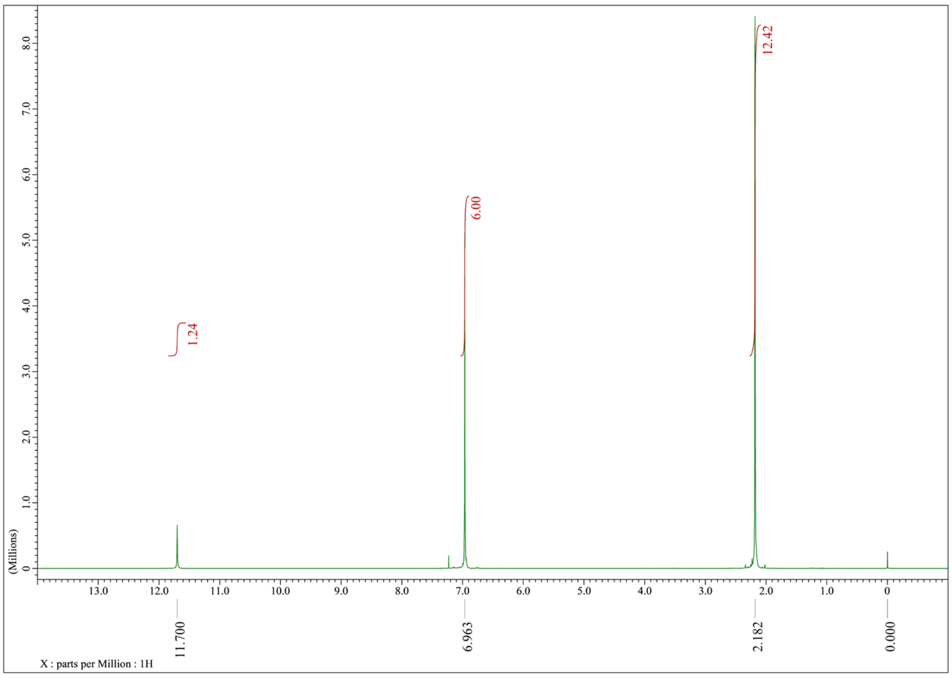

^{13}C NMR (100 MHz, $CDCl_3$).

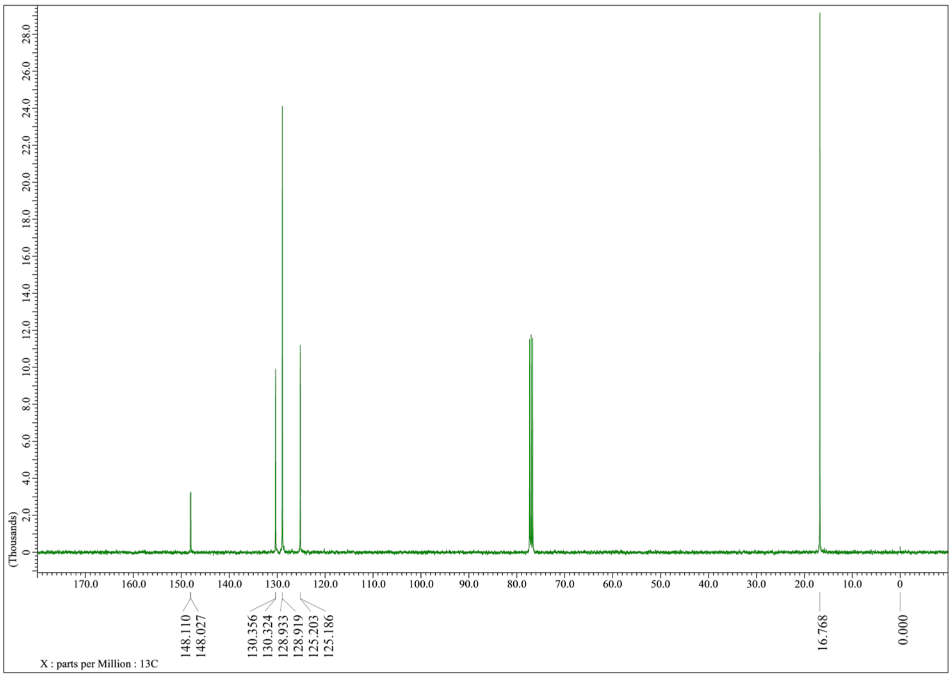

^{31}P NMR (162 MHz, $CDCl_3$).

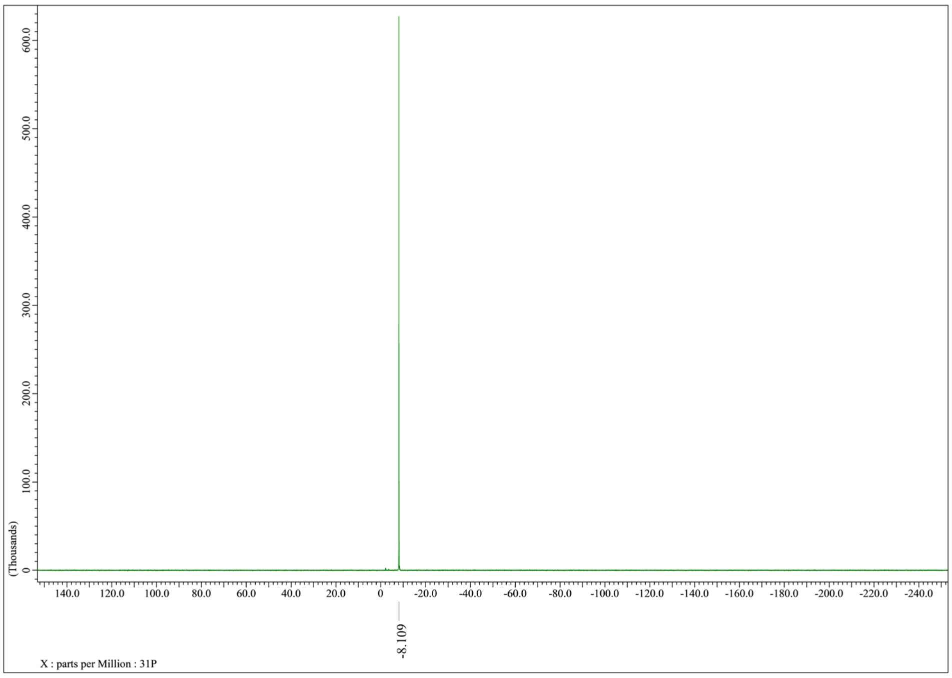

*1.4.4. NMR spectra for synthesized standards: bis(4-cyanophenyl)phosphate (**2j**)*

^{1}H NMR (400 MHz, D_2O).

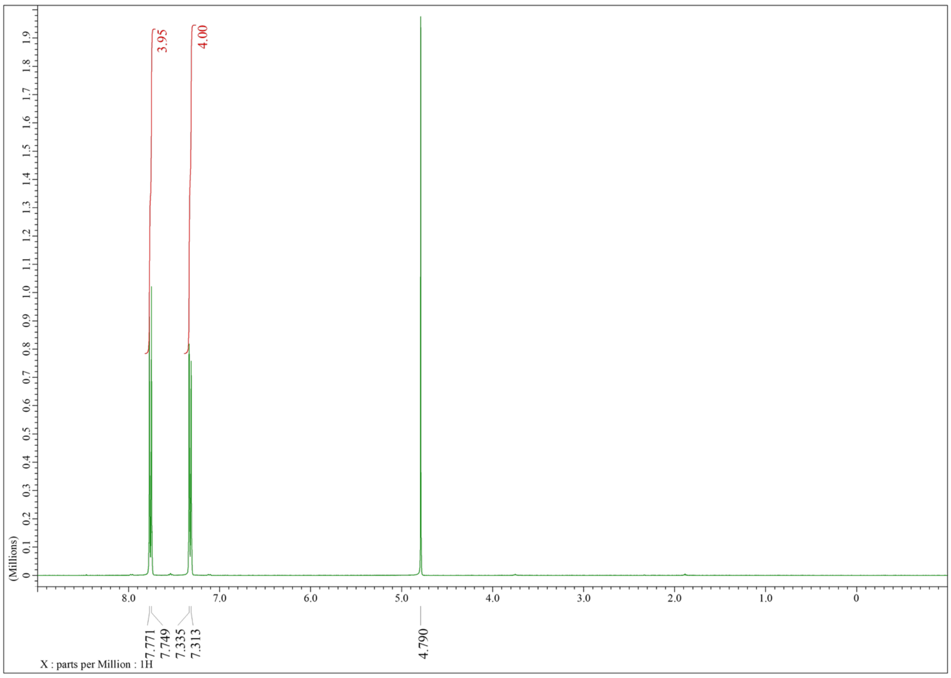

^{13}C NMR (100 MHz, D_2O).

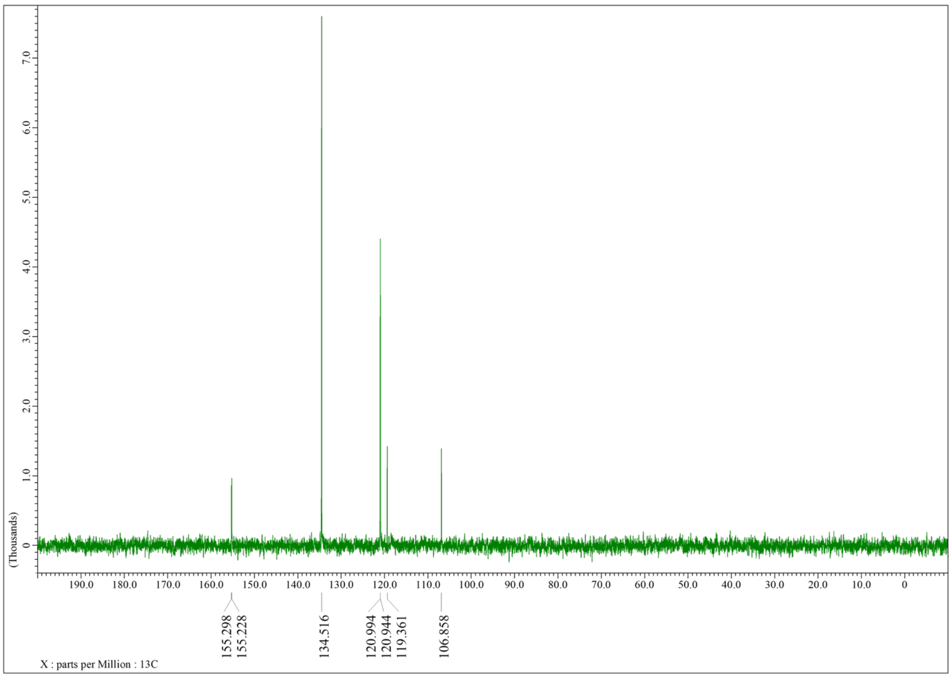

^{31}P NMR (162 MHz, D_2O).

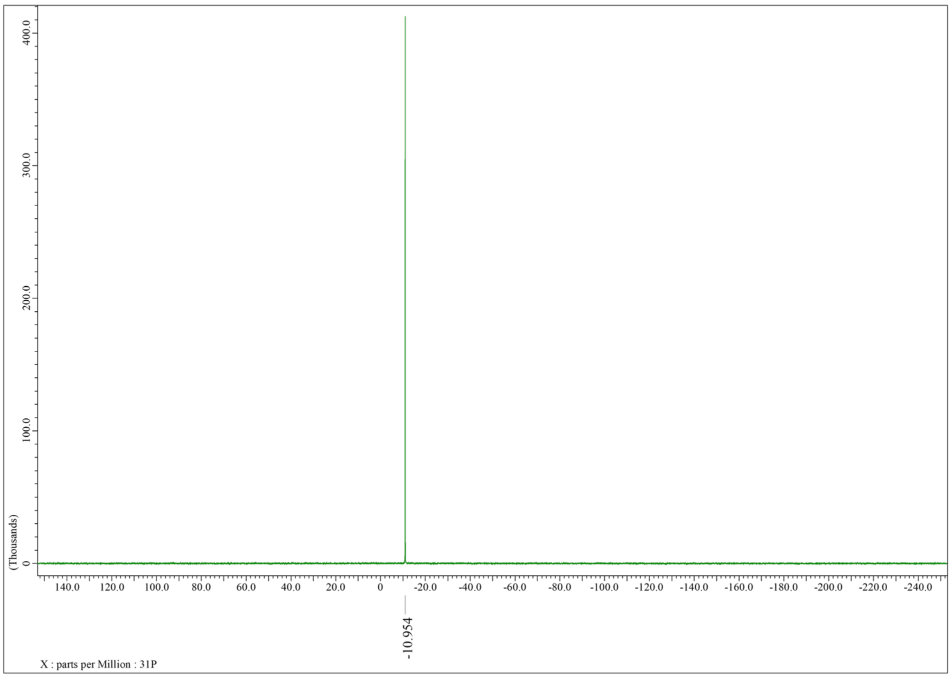

2. A time course analysis of phosphate ester synthesis

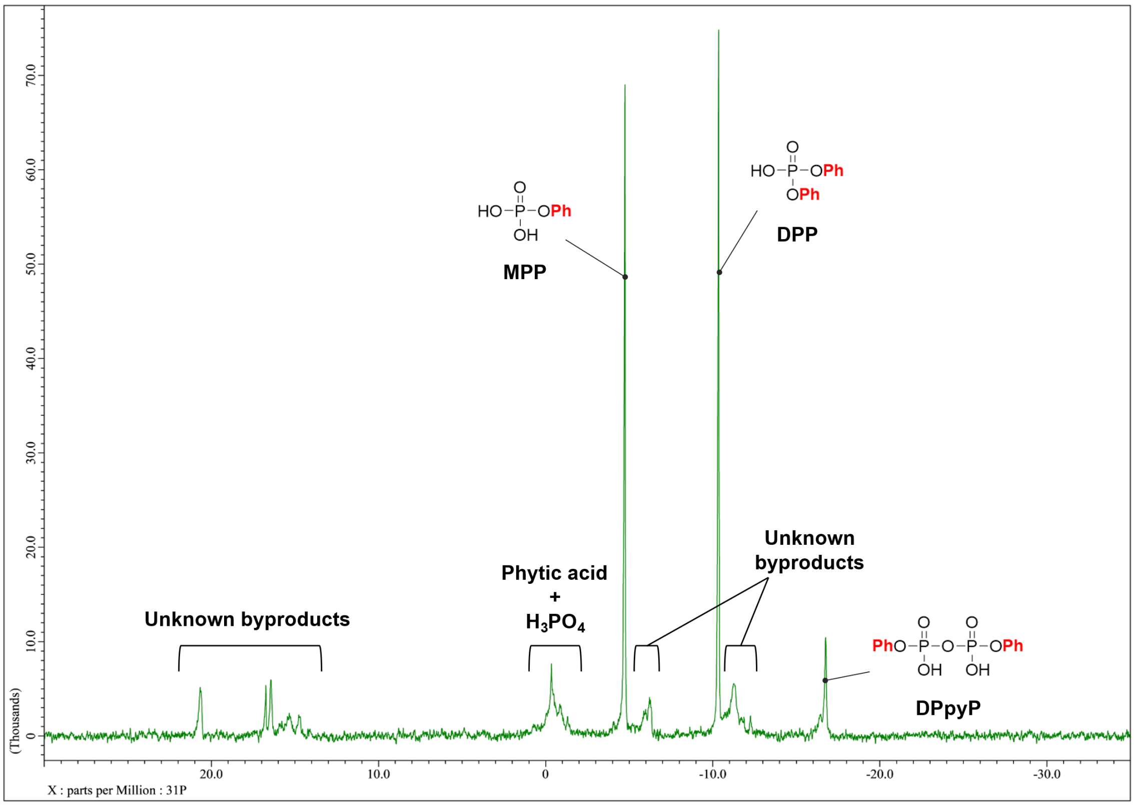

Figure S2. ^{31}P NMR spectrum of the reaction mixture obtained at 24 h during the time-course analysis.

According to the ^{31}P NMR spectrum and chemical shifts, it was considered that a diphenyl pyrophosphate (DPpyP) was formed [5].

3. ^{31}P NMR spectrum for commercial and extracted phytic acid

3.1. NMR spectra for extracted phytic acid (solvent: D_2O)

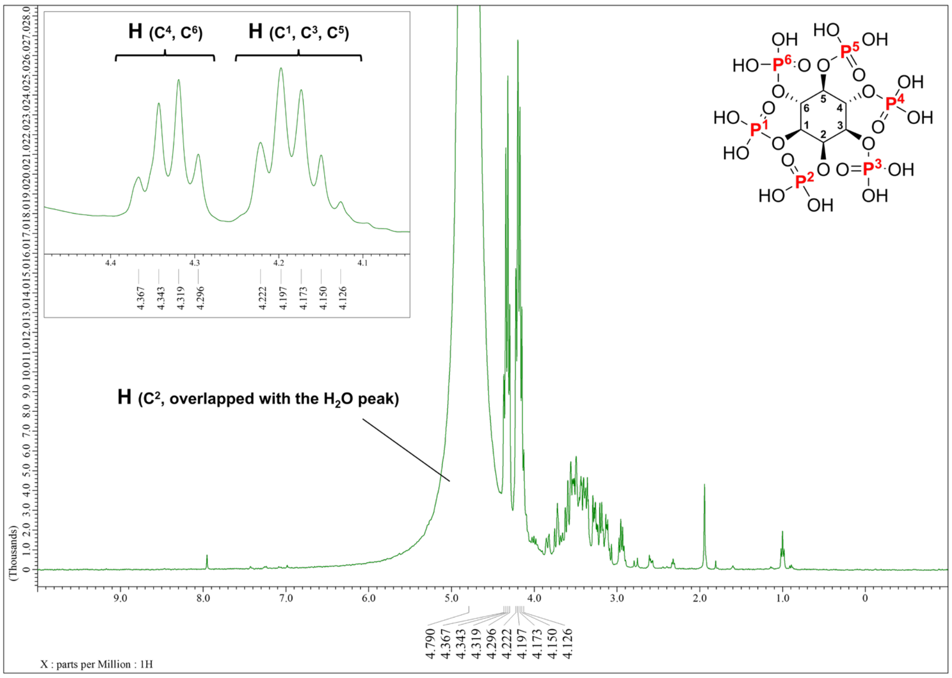

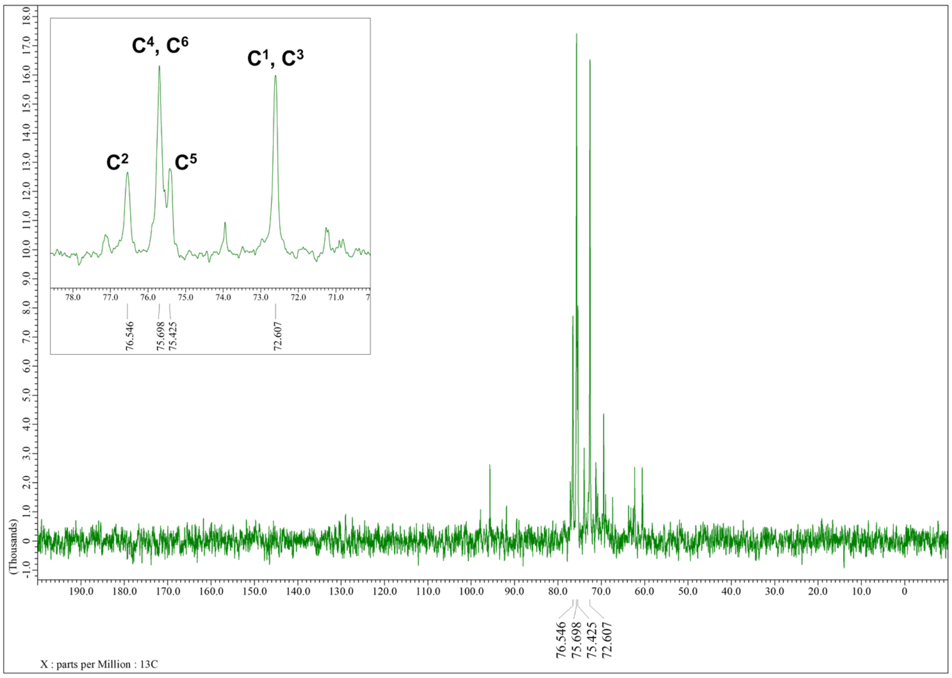

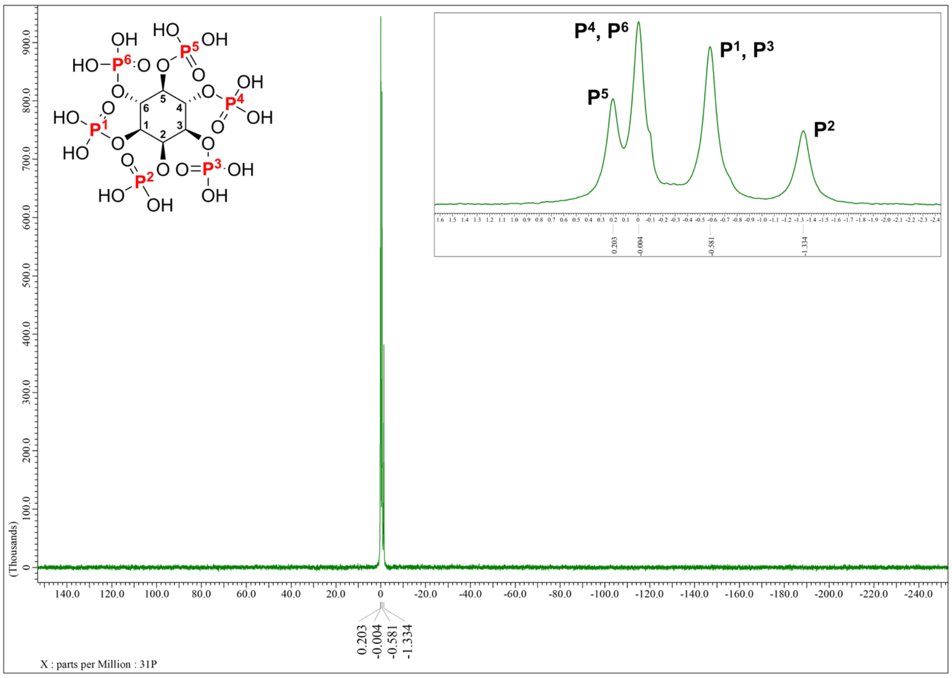

*The peaks assignments were based on references [6-8]

3.2. Comparison of NMR spectra between extracted and commercial phytic acid

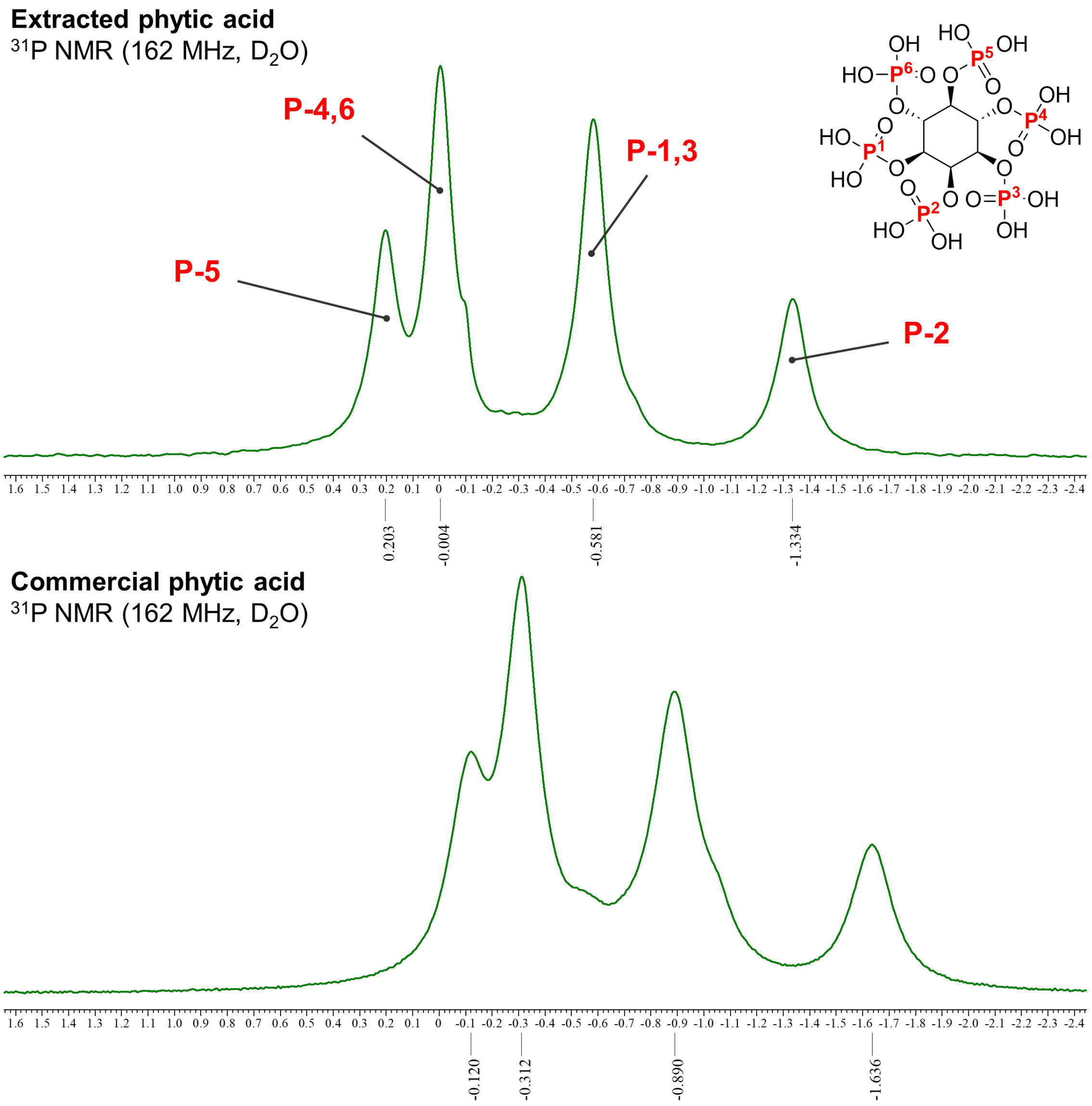

Figure S3. ^{31}P NMR spectrum of phytic acid extracted from rice bran.

The extracted phytic acid solution was diluted with D_2O, and ^{31}P NMR was measured with ^{1}H decoupling. Detected phytic acid peaks were compared with the previous studies and agreed with the references [6-8].

4. Phosphate ester synthesis using extracted phytic acid

*4.1. Synthesis method of **2a** using extracted phytic acid*

The reaction apparatus was set on the oil bath and N_2 flow was set to 0.1 L/min. Extracted phytic acid (0.7 mmol, 3.7 M aqueous solution) was added to a DMF (6 mL). Triethylamine (12.6 mmol) and **1a** (42 mmol) were poured into the mixture. The oil bath temperature was set to 200 °C and the reaction was carried out for 48 hours under mixing with magnetic stirring. After the reaction was completed, the excess amount of **1a** and DMF were removed by the vacuum distillation by using Kugelrohr apparatus set to 160 °C. The residue was dissolved with toluene (30 mL) and deionized water (27 mL), and each solution was filtered to remove black burnt. Then two filtrates were mixed, and conc. HCl (3 mL) was added to the mixture. The mixture was well shaken, and the organic layer was collected. The collected layer was washed with 1.2 M HCl (30 mL) again. A few drops of 0.1% phenolphthalein (dissolved in 90% ethanol) were added into the collected organic layer, and the organic layer was neutralized with 1.0 M NaOH (methanol solution). The solution was evaporated, and the precipitate was filtered by vacuum filtration and washed with toluene. The residue was dried under vacuo to obtain a sodium diphenylphosphate (**2a**) as a light brown solid; 500 mg (1.84 mmol, 44%); ^{1}H NMR (400 MHz, D_2O): δ 7.39 (t, 4H, J = 8.0 Hz), 7.23–7.19 (m, 6H) ppm; ^{13}C NMR (100 MHz, D_2O): δ 151.7 (d, $J_{C–P}$ = 7.3 Hz), 129.8, 124.5, 120.2 (d, $J_{C–P}$ = 4.5 Hz) ppm; ^{31}P NMR (162 MHz, D_2O): δ -8.89 ppm; MS (ESI) $[M–Na]^-$ m/z = 249; m.p.: 212–214 °C.

The spectral data were consistent with previously reported data [1,2].

*4.2. NMR spectra for diphenyl phosphate (**2a**) synthesized from extracted phytic acid,*

^{1}H NMR (400 MHz, D_2O).

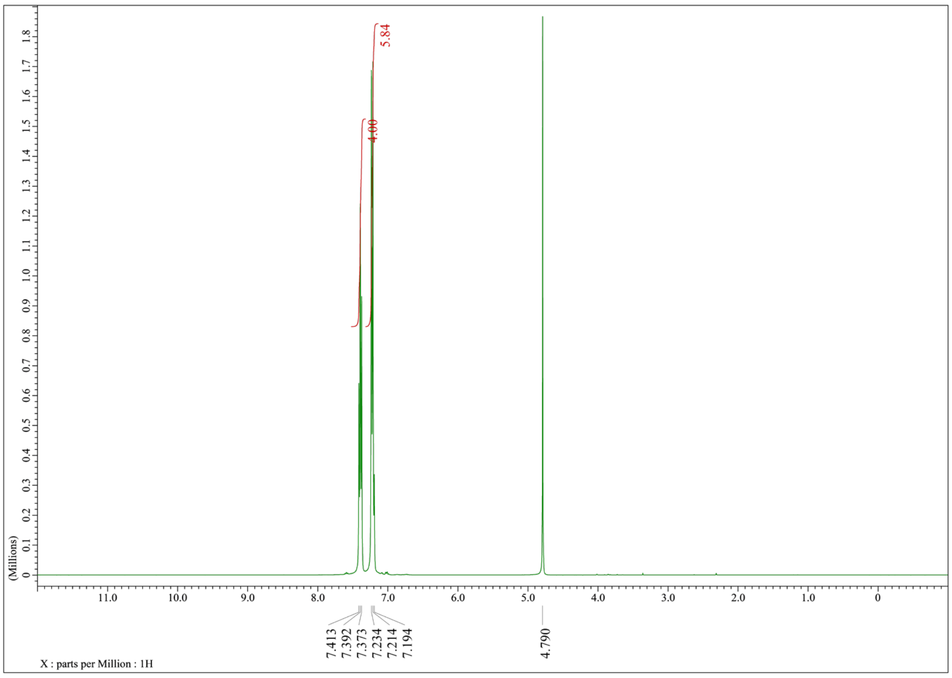

^{13}C NMR (100 MHz, D_2O).

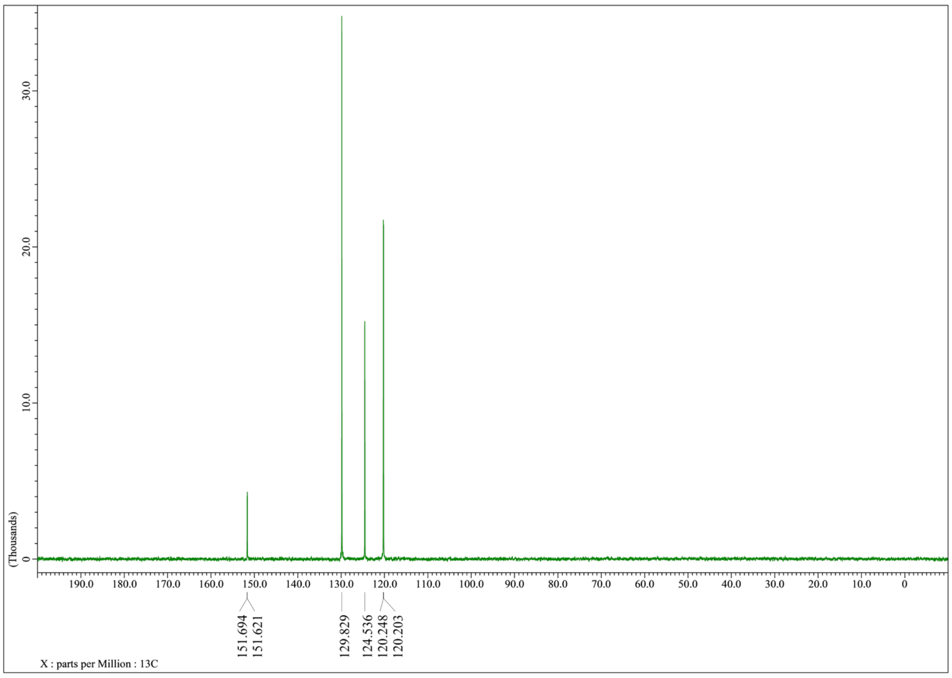

^{31}P NMR (162 MHz, D_2O).

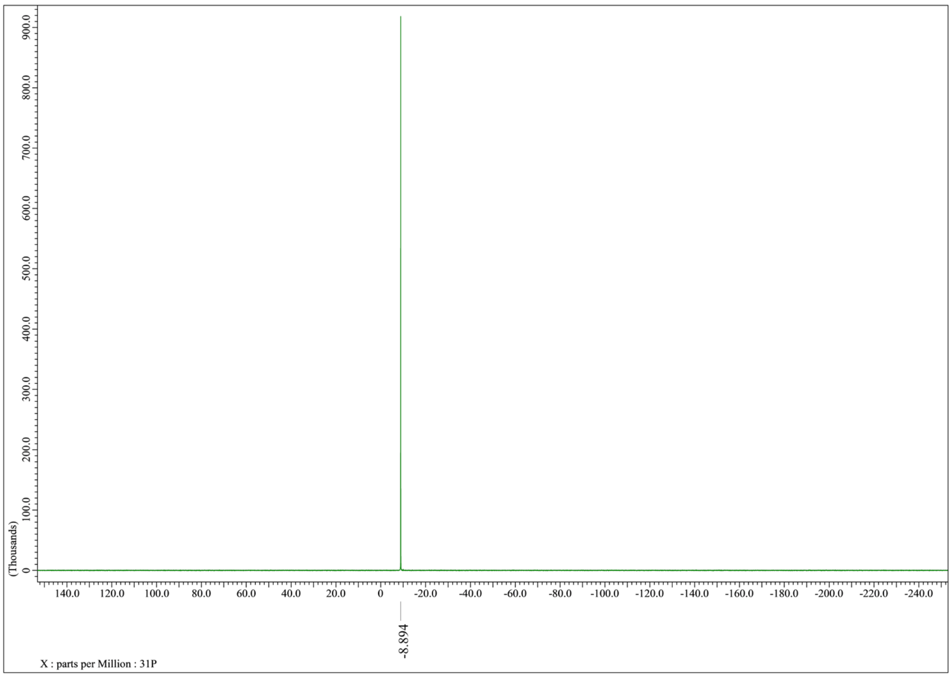

*4.3. HPLC spectrum for sodium diphenylphosphate (**2a**) derived from extracted phytic acid*

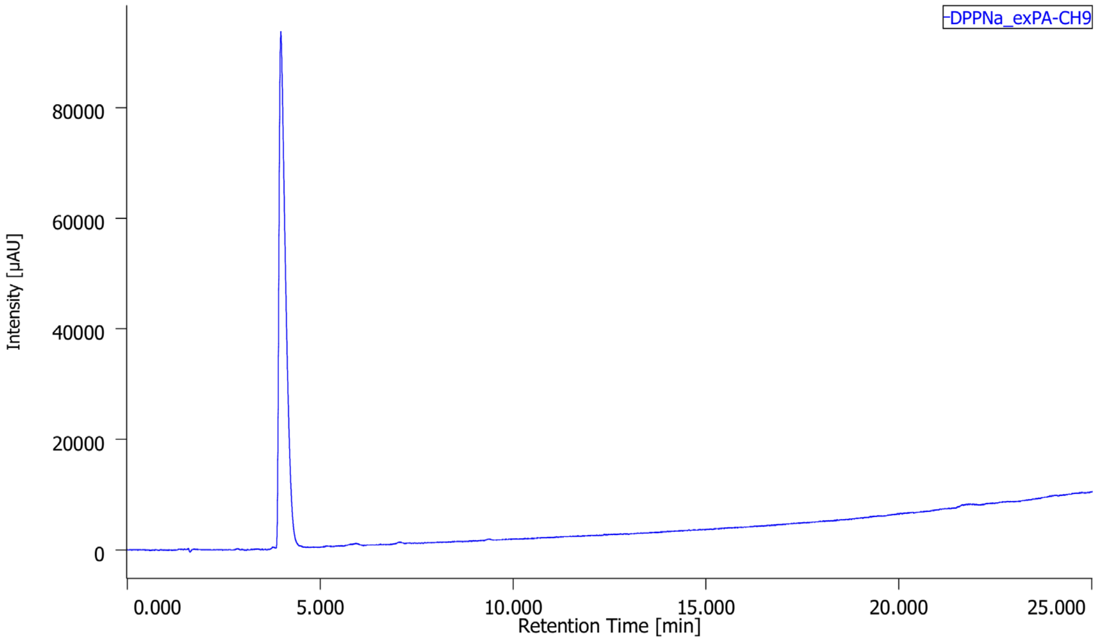

Figure S4. HPLC spectrum of **2a**, which was synthesized from the extracted phytic acid.

The purity of **2a** derived from the extracted phytic acid was greater than 95%. Analysis conditions were as follows.

Instruments	LC-2000Plus series (Jasco, Tokyo, Japan)
Column	Inertsil ODS-3 (4.6 mm x 150 mm; particle size: 5 μm; GL Sciences, Tokyo, Japan)
Temperature	40 °C
Mobile phase	0.1% trifluoroacetic acid in both water (A) and methanol (B)
Gradient elution	from 60% to 100% B (0–25 min)
Flow rate	1.0 mL/min
Detection	photodiode array detector set to 254 nm
Injection volume	10 μL (1 mg/mL of **2a** in methanol)

5. References

1. Tran, C. C.; Asao, K.; Sasaki, T.; Hayakawa, Y.; Kawaguchi, S. *Tetrahedron Lett.* **2022**, *96*, 153726. doi:10.1016/j.tetlet.2022.153726

2. Macdonald, E. K.; Shaver, M. P. *Eur. Polym. J.* **2017**, *95*, 702–710. doi:10.1016/j.eurpolymj.2017.04.005

3. Kim, S.; Park, H.; Fuß, F.; Lee, Y. *Polym. Chem.* **2023**, *14*(21), 2610–2616. doi.org/10.1039/D3PY00239J

4. Chen, L.-H.; Zhang, Q.; Xiao, Y.-F.; Fang, Y.-C.; Xie, X.; Nan, F.-J. *J. Med. Chem.* **2022**, *65*, 3991–4006. doi:10.1021/acs.jmedchem.1c01813

5. Haas, T. M.; Wiesler, S.; Dürr-Mayer, T.; Ripp, A.; Fouka, P.; Qiu, D.; Jessen, H. J. *Angew. Chem. Int. Ed.* **2022**, *61*, e202113231. doi.org/10.1002/anie.202113231.

6. Makarenko, N. V.; Zemnukhova, L. A.; Nemtarev, A. V.; Kovekhova, A. V.; Arefieva, O. D. *Bioresour.* **2018**, *13*, 3411–3419. doi:10.15376/biores.13.2.3411-3419

7. Bebot-Brigaud, A.; Dange, C.; Fauconnier, N.; Gérard, C. *J. Inorg. Biochem.* **1999**, *75*, 71–78. doi.org/10.1016/S0162-0134(99)00041-0.

8. Veiga, N.; Torres, J.; Macho, I.; Gómez, K.; González, G.; Kremer, C. Coordination. *Dalton Trans.* **2014**, *43*, 16238–16251. doi.org/10.1039/C4DT01350F.
